# Understanding the Thickness and Light-Intensity Dependent Performance of Green-Solvent Processed Organic Solar Cells

**DOI:** 10.1021/acsmaterialsau.2c00070

**Published:** 2023-01-25

**Authors:** Dana Lübke, Paula Hartnagel, Markus Hülsbeck, Thomas Kirchartz

**Affiliations:** † IEK5-Photovoltaics, Forschungszentrum Jülich, 52425 Jülich, Germany; ‡ Faculty of Engineering and CENIDE, University of Duisburg-Essen, Carl-Benz-Str. 199, 47057 Duisburg, Germany

**Keywords:** organic photovoltaics, OPV, low light, indoor, shunt resistance, ideality factor

## Abstract

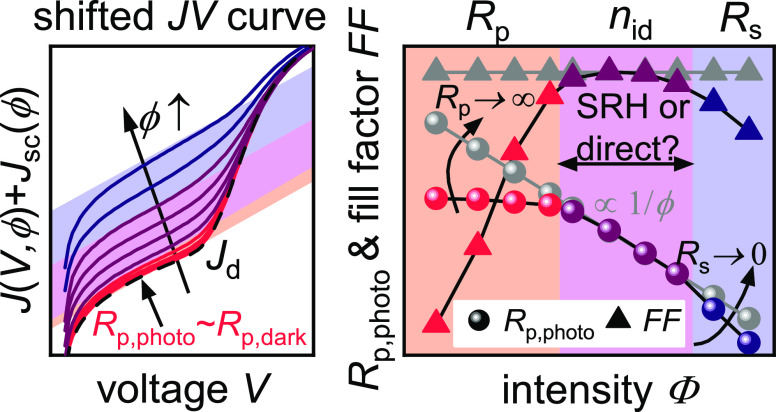

For indoor light harvesting, the adjustable band gap of molecular semiconductors is a significant advantage relative to many inorganic photovoltaic technologies. However, several challenges have to be overcome that include processability in nonhalogenated solvents, sufficiently high thicknesses (>250 nm) and high efficiencies at illuminances typically found in indoor environments. Here, we report on the development and application of new methods to quantify and identify performance losses based on thickness- and intensity-dependent current density–voltage measurements. Furthermore, we report on the fabrication of solar cells based on the blend PBDB-T:F-M processed in the nonhalogenated solvent *o*-xylene. In the low-intensity regime, insufficiently high shunt resistances limit the photovoltaic performance and by analyzing current density voltage–curves for solar cells with various shunt resistances we find that ∼100 kΩ cm^2^ are required at 200 lux. We provide a unified description of fill factor losses introducing the concept of light-intensity-dependent apparent shunts that originate from incomplete and voltage-dependent charge collection. In experiment and simulation, we show that good fill factors are associated with a photo-shunt inversely scaling with intensity. Intensity regions with photo-shunt resistances close to the dark-shunt resistance are accompanied by severe extraction losses. To better analyze recombination, we perform a careful analysis of the light intensity and thickness dependence of the open-circuit voltage and prove that trap-assisted recombination dominates the recombination losses at low light intensities.

## Introduction

The efficiencies of organic solar cells have rapidly increased over the last years thanks to the development of well-absorbing new donor and acceptor molecules. Many of the fundamental disadvantages of organic photovoltaics such as the relatively low open-circuit voltage due to the donor–acceptor offset are mitigated to an extent^1^ that 1 sun efficiencies close to 20% are now possible.^2−11^ For transfer of the technology to market, however, various technological and economic challenges have to be overcome.^12−14^ Particular concerns are the ability to use roll-to-roll deposition in air using green solvents,^15^ employing considerable active-layer thicknesses in the ∼300 nm range^16−18^ and to achieve sufficient efficiency and stability^19,20^ for a certain application. As this is currently still difficult for mainstream applications of solar modules on roofs or solar parks, alternative applications have become more important for start-ups and scientific research. These applications include the use of small solar modules for indoor applications and the internet of things.^21,22^ Here, smaller modules are needed that operate at low illumination intensities around 200 lux and power, e.g., a sensor combined with some communication unit.

In addition to technological and material science challenges, the development of organic photovoltaics for indoor applications also requires methodological innovations to better characterize and understand the major loss mechanisms and their dependence on light intensity. It is highly beneficial if characterization of the solar cells can be done from indoor illumination conditions (∼200 lux intensity created with the spectrum of a light emitting diode (LED)) to 1 sun outdoor conditions as performance-determining loss mechanisms change with light intensity. Covering a large range in illumination conditions with a single light source (such as an LED) is typically difficult. Changing the LED driving current will allow changing the intensity in a certain range, while further variations in intensity will have to be dealt with by varying the working distance between light source and cell or by using neutral density (ND) filters. The first option is not always feasible for space reasons, and the second option suffers from the wavelength-dependent transmission that is unavoidable in ND filters. Finally, the comparison between the quality and efficiency of organic solar cells for indoor use suffers from the lack of standards and the high differences in efficiency as a function of the exact illuminance and LED spectrum.^23^ Thus, there are few publications in the field of organic photovoltaics that cover and explain device performance over a large range of light intensities and that provide data that is widely comparable with other efforts in the field.

Here, we present an attempt to tackle a range of the above-mentioned challenges. We develop a process to fabricate PBDB-T:F-M organic solar cells using the solvent *o*-xylene, which is a nonhalogenated solvent useful for industrial applications. In order to investigate the feasibility of going toward larger active-layer thicknesses, we perform several thickness series on different device areas (*A* = 0.06 cm^2^ and *A* = 0.16 cm^2^) and perform intensity-dependent measurements from 200 lux to 1 sun on each of the cells. We achieve a variation in LED intensity using a combination of ND filters and changing the LED driving current and illustrate the effect of the nonconstant transmission of typical ND filters.

The combination of thickness and intensity-dependent data allows us to quantify several loss mechanisms, namely, losses due to imperfect extraction and recombination. For different intensity regimes, the most important performance-limiting mechanisms will change. At lower light intensities, shunt resistances become relatively more important than series resistances and primarily determine extraction losses, quantified in reduced open-circuit voltages (*V*_oc_) and fill factors (FF). In order to understand the effect of shunt resistances on the device performance, we modulate the shunt resistance by changing the active-layer thickness. We analyze our data according to critical shunt resistances, above which good photovoltaic performance is maintained in the low light regime. However, the organic solar cells studied in this work show different FF values at a constant shunt resistance, meaning that the sole consideration of the dark-shunt resistance fails to explain extraction losses under illumination. Hence, in this study, we introduce the concept of the photo-shunt resistance, which is an apparent shunt under illumination and with which we can explain extraction losses at different light intensities. In the mid-intensity range (once a sufficient shunt resistance is ensured), photovoltaic performance is mainly limited by the decrease of *V*_oc_ with intensity, which is determined by the ideality factor. Therefore, we analyze the ideality factor at different light intensities and draw conclusions regarding the underlying recombination mechanism. In addition, we determine if recombination is predominantly occurring in the bulk or at the contacts by examining the saturation-current density at open circuit and extend this method to intensity-dependent data. Our study shows that thickness and intensity-dependent data sets are highly valuable and shed light on different loss mechanisms determining the performance in the low light regime.

## Results and Discussion

### Donor–Acceptor Blend

In order to establish organic photovoltaics for industrial low light applications, the donor–acceptor blend has to satisfy certain requirements. In this study, we chose PBDB-T as the donor and the small molecule F-M as the acceptor (exact name can be found in the Methods section). Zhao et al. showed that PBDB-T is soluble in different halogen-free solvents.^24^ We developed a process for dissolving PBDB-T:F-M in the industrial-friendly solvent *o-*xylene and also tested the process working under ambient conditions without severe performance losses. In addition to the production-related requirements, the overlap of the materials’ absorption (defined by the band gap *E*_g_) and the LED spectrum is of great importance. Recently, we made calculations of the maximum efficiency of indoor solar cells using a modified Shockley–Queisser (SQ) model, where we used LED spectra at various intensities instead of a solar spectrum. Unsurprisingly, the optimum efficiency and band gap at a constant illuminance depends on the color temperature and generally the illumination spectrum of the used LED. While for cold white LEDs maximum efficiencies of 54% are reached for materials with band gaps of ∼2 eV, for warm white LEDs, the optimal band gap is around 1.7–1.8 eV.^23^ Furthermore, we showed that the maximum output-power density *P*_out,SQ_ at a constant illuminance can be increased by using warm white LEDs compared to neutral white LEDs (e.g., the maximum *P*_out,SQ_ for a 4100 K LED at 200 lux is 26 μW/cm^2^ compared to a *P*_out,SQ_ of 36 μW/cm^2^ with an 2700 K LED).^23^ As will be discussed later, once sufficiently high parallel resistances are ensured, mainly the *V*_oc_ decrease with decreasing light intensity is determining the efficiency,^25^ which highlights the importance of materials with high band gaps. In addition, the *V*_oc_ is decreasing faster with light intensity if trap-assisted recombination is dominant (ideality factor *n*_id_ ≈ 2). Hence, good material quality should be ensured.

**Figure 1:**
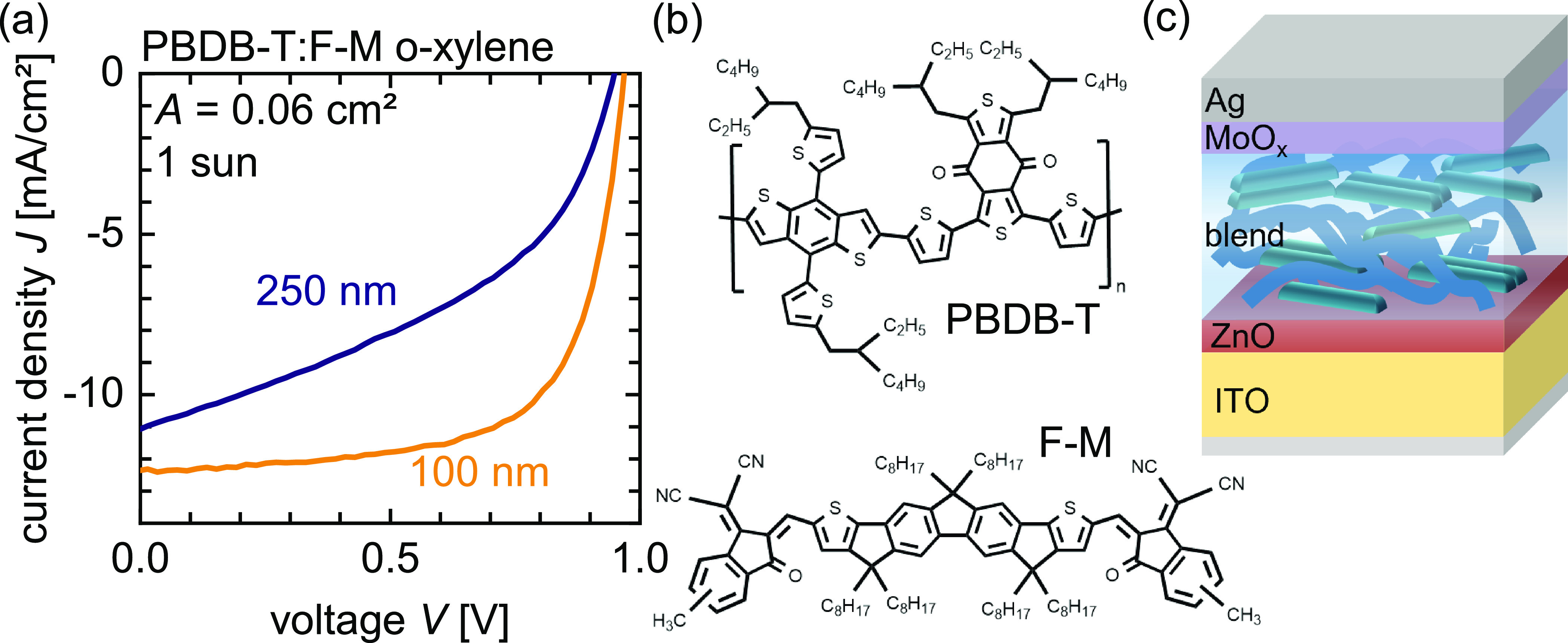
(a) Current density–voltage curves of the PBDB-T:F-M devices processed in *o*-xylene with an active area of *A* = 0.06 cm^2^ and absorber thicknesses of 100 and 250 nm. (b) Chemical structure of the PBDB-T and F-M molecules and (c) used inverted device structure ITO/ZnO/blend/MoO_*x*_/Ag.

The highest occupied molecular orbital (HOMO) and the lowest unoccupied molecular orbital (LUMO) of PBDB-T are −5.3 and −3.5 eV^26^ and those of F-M are at −5.42 and −3.7 eV, respectively.^27^ Recently, PBDB-T:F-M was used as a front cell in tandem^27,28^ and multijunction^26^ solar cells. Firdaus et al. reached an open-circuit voltage *V*_oc_ of 0.98 V, a short-circuit current density *J*_sc_ of 15.4 mA/cm^2^, and a fill factor FF of 71.7%, which results in an efficiency η of 10.9% under 1 sun conditions.^26^ In Figure 1, the current density–voltage (*J*–*V*) curves of the ITO/ZnO/PBDB-T:F-M/MoO_*x*_/Ag devices (chemical names can be found in the Methods section) are shown with absorber thicknesses of ∼100 and ∼250 nm processed in *o*-xylene. With a *V*_oc_ of 0.97 V, a short-circuit current density *J*_sc_ of 12.95 mA/cm^2^, and a fill factor FF of 67.7%, the thin device reaches an efficiency η of 8.4% under 1 sun conditions. This value is slightly lower than the device of Firdaus et al. but is processed in *o*-xylene and without the additional interlayer PFN-Br used by Firdaus et al. While this material combination shows average performance under 1 sun, the high band gap of 1.7 eV (which is optimal for a 2700 K LED, see Figure S1) and the strong absorption between 450 and 710 nm^26^ promise high *P*_out_ and high efficiencies for the low light regime.

### Thickness- and Intensity-Dependent Device Performance

In order to scan solar cell performance from the low light regime to 1 sun, a wide range of intensities has to be covered. In the Influence of the LED Spectrum on Device Performance section, we ensure defined measuring conditions by examining the effect of varying ND Filters on the output-power density and efficiency. In the low light regime, device performance is limited by leakage currents through shunt resistances. In the Impact of Resistive Elements on Intensity-Dependent Device Performance section, we present the analysis of thickness- and intensity-dependent current density–voltage (*J* – *V*) curves and focus on the influence of resistive elements on the device performance in the low-intensity regime. In the mid-intensity range, device performance is determined by the logarithmical *V*_oc_ decrease with intensity. For Shockley–Read–Hall (SRH) recombination (ideality factor *n*_id_ ≈ 2), the *V*_oc_ decreases faster with intensity compared to direct recombination. Thus, device performance is determined in the mid-intensity range by the underlying recombination mechanism. Therefore, in the Determining Recombination Mechanisms with Intensity- and Thickness-Dependent Data section, we examine the ideality factor and the saturation-current density at open circuit to identify the dominant recombination mechanism.

#### Influence of the LED Spectrum on Device Performance

**Figure 2:**
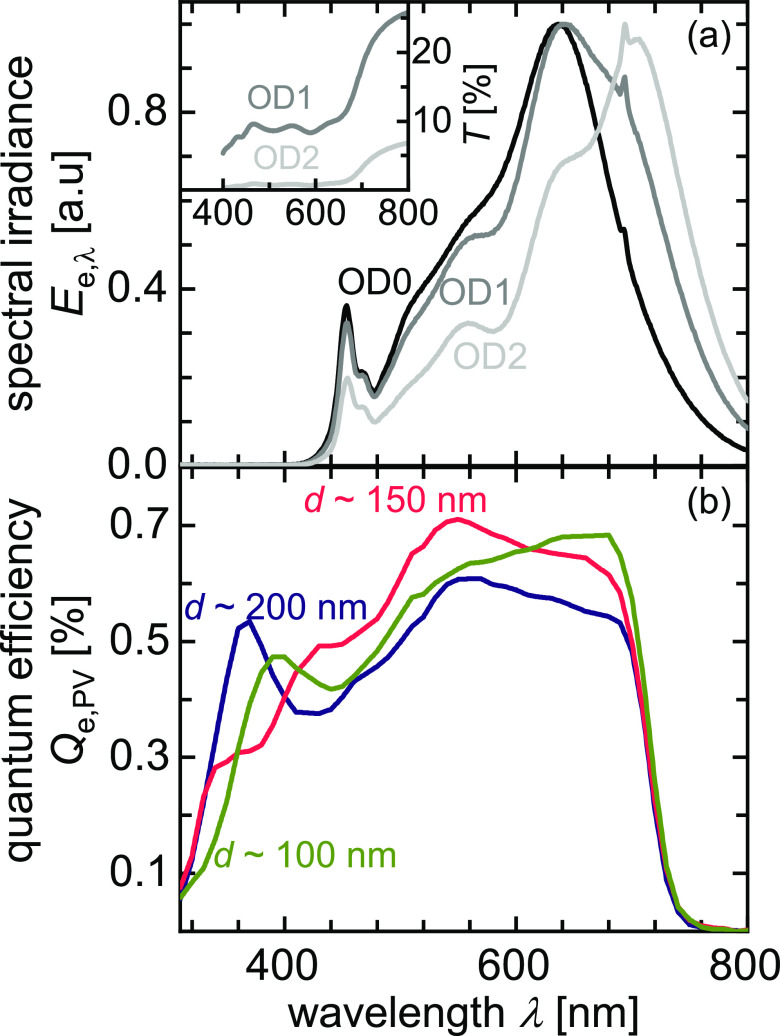
(a) Normalized spectral irradiance of a 2700 K LED (depicted in black, OD 0) and with the used OD 1 filter (depicted in dark gray) and OD 2 filter (depicted in light gray). The wavelength-dependent transmission of the used ND filters (given by the manufacturer) is depicted in the inset and causes a small red shift for the OD 1 and a stronger red shift for the OD 2 filter. (b) Quantum efficiency of PBDB-T:F-M samples with different thicknesses and an active area of *A* = 0.16 cm^2^.

As the performance in the low light regime depends on the intensity, we need to define our measuring conditions properly before we can discuss the performance itself. For this, we performed absolute spectral irradiance measurements with an integrating sphere of the LED light source in combination with several ND filters. We then performed intensity-dependent measurements of the *J*–*V* curves as well as quantum-efficiency measurements of the solar cells and used the data to accurately determine the output-power density and the efficiency at different illuminances or irradiances. The light intensity was varied by a combination of ND filters and changing the LED current in the linear range of LED driving current vs LED output-power density. The spectral irradiance *E*_e,λ_ of a 2700 K LED, as well as the spectrum of the same LED with an OD 1 filter (dark gray) and an OD 2 filter (light gray), is depicted in Figure 2a. The inset in Figure 2a shows the transmission data of the ND filters (according to manufacturer information), which are more or less constant in the region λ < 650 nm and are strongly increasing for higher wavelengths. As a result, the spectral irradiance with the OD 1 filter is slightly red-shifted, and the spectrum with the OD 2 filter is strongly red-shifted. This red shift and the decrease in transmission affect the output-power density *P*_out_, the input-power density *P*_in_, and therefore the efficiency η = *P*_out_/*P*_in_ of solar cells.

First, the input-power density *P*_in_ (or irradiance *E*_e_) is obtained by integrating the spectral irradiance *E*_e,λ_ (usually given in μW/cm^2^/nm)
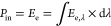
1

Second, the output-power density is determined by the *J*_sc_, the *V*_oc_, and the FF as *P*_out_ = *J*_sc_*V*_oc_FF. The *J*_sc_ is determined by
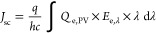
2where *q* is the elementary charge, *h* is Planck’s constant, *c* is the speed of light, and *Q*_e,PV_ is the quantum efficiency of the solar cell, which can be found in Figure 2b. From these equations, it is clear that a change in the LED spectra by using ND filters will strongly affect photovoltaic performance. For indoor applications, photovoltaic performance is measured with photometric quantities to compare the brightness perceived by a human observer rather than a standardized power density *E*_e_. The photometric equivalent, the illuminance *E*_v_ given in lux, is obtained by integrating the product of the spectral irradiance with the standard sensitivity curve *V*(λ) of the human eye and the coefficient *K*_m_= 638 lmW^–1^ as described by
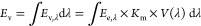
3

The illuminance *E*_v_ is the third quantity affected by the spectrum change caused by the ND filters. Although the use of ND filters is a practical way to change the intensity over a broad range, the wavelength-dependent transmission complicates data analysis, due to its high impact on photovoltaic performance.

**Figure 3:**
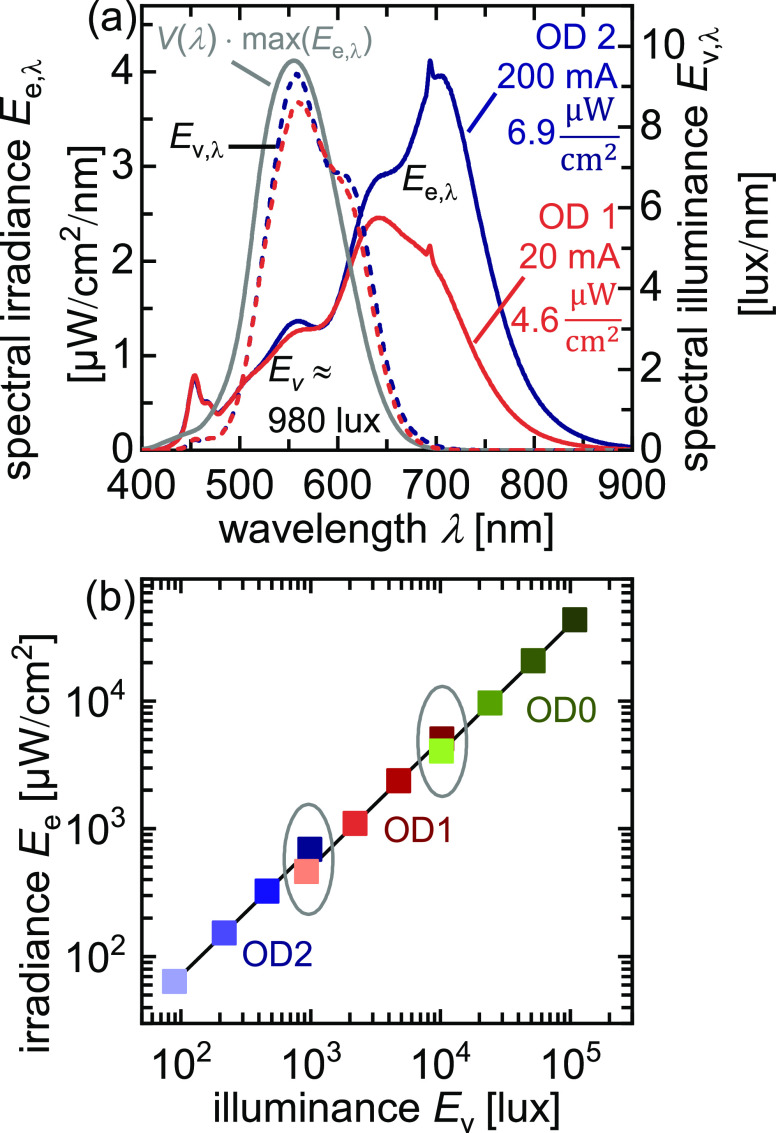
(a) Absolute spectral irradiance *E*_e,λ_ (solid lines, left axis) of the used LED with an OD 2 filter operated at a LED current of 200 mA (blue) and with an OD 1 filter with 20 mA (bright red). The absolute spectral illuminance is shown in dashed lines on the right axis. The gray solid line depicts the normalized *V*(λ) curve with the maximum at 555 nm. The spectrum with the OD 2 filter is red shifted, so that a higher input-power density is needed to reach the same illuminance compared to OD 1 filter. (b) Irradiance *E*_e_ plotted against the illuminance *E*_v_ for all currents and filters used. The filter change shown in (a) causes a jump to lower irradiances, although the illuminance remains constant.

In our experiments, we used four LED currents *I*_LED_ (20, 45, 95, and 200 mA) with an OD 2 and OD 1 and without a ND filter. As can be seen in Figure S2, the relative spectral irradiances remain constant over the whole range of *I*_LED_. To examine the effects of changing filters, we chose LED currents, so that, for example, the 200 mA measurement with the OD 2 filter is resulting in the same illuminance as the 20 mA measurement with the OD 1 filter. The absolute spectral irradiance and the absolute spectral illuminance of this explicit situation are depicted in Figure 3a, and the irradiances of the whole measuring range are plotted against the illuminance in Figure 3b (the listed parameters can be found in Table S1).

**Figure 4:**
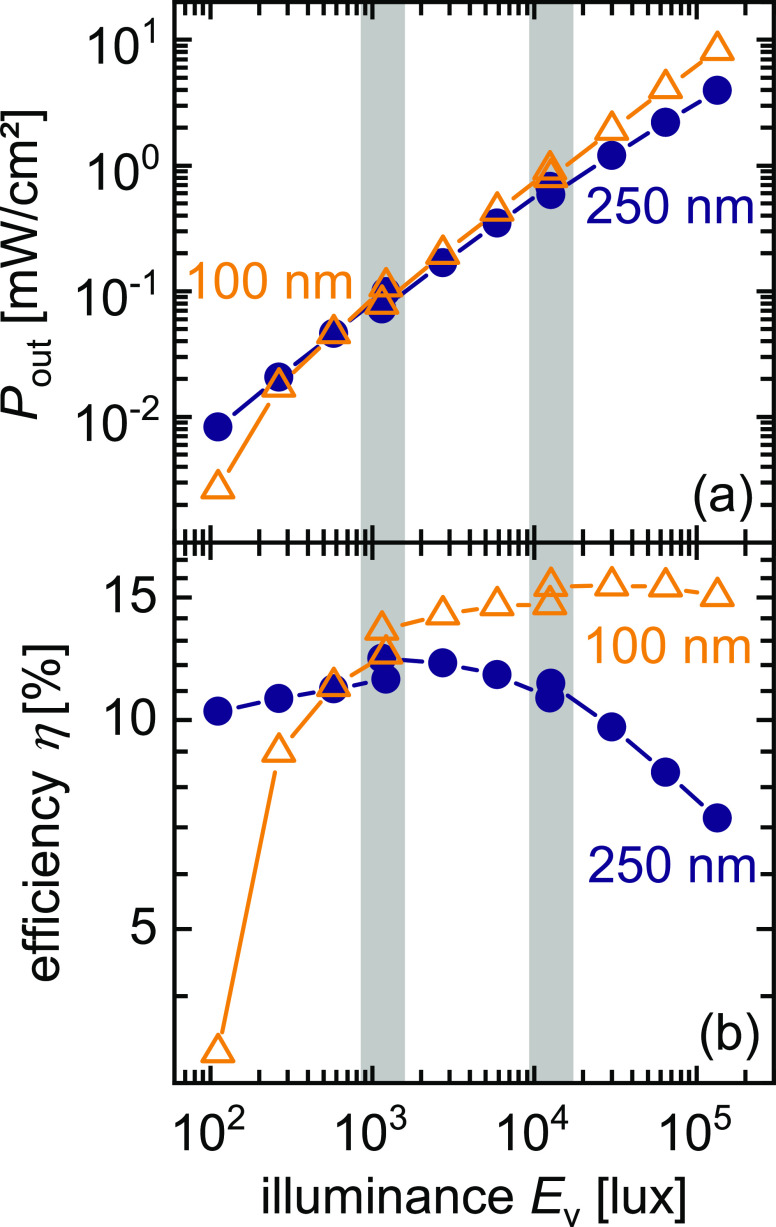
(a) Output-power density *P*_out_ and (b) efficiency of two PBDB-T:F-M organic solar cells with different thicknesses and a device area of *A* = 0.06 cm^2^. Illuminances affected by the filter change are highlighted with gray squares.

The spectral illuminances (dashed lines, Figure 3a) of both curves overlap, indicating a constant illuminance ∼980 lux. As the *E*_e,λ_ spectrum originating from the OD 2 filter (blue solid line) is strongly red shifted and thus further away from the *V*(λ) curve, there is less usable light to the human eye and more intensity is needed to reach the same illuminance compared to the less red shifted spectrum with the OD 1 filter (solid red line). As a result, the *E*_e,λ_ spectra of the 200 mA OD 2 measurement exhibit an input-power density of 6.9 μW/cm^2^, whereas the 20 mA OD 1 spectrum gives an input-power density of 4.6 μW/cm^2^. Therefore, if measuring from low to high LED currents, a change of filters results in an abrupt decrease of the input-power density at the same illuminance (Figure 3b). The output-power density *P*_out_ and the efficiency η of PBDB-T:F-M organic solar cells with an absorber thickness of 100 and 250 nm are plotted versus the illuminance in Figure 4. According to eqs 1 and 2, the decrease of the input-power density due to the filter change will cause a decrease in the *J*_sc_ and therefore *P*_out_ is abruptly deceasing and the efficiency is abruptly increasing (gray area in Figure 4a,b).

The device performance depends on the overlap of the solar cells *Q*_e,PV_ and the *E*_e,λ_ of the used LED. Recently, we reported a method, which enables us to scan the performance for different LED spectra at a certain illuminance if the *Q*_e,PV_ and the *E*_e,λ_ are known.^23^ The performance parameters are plotted against the color temperature of various LEDs in Figure S1, showing that PBDB-T:F-M devices reach maximum efficiencies for 2700 K LEDs. As can be seen from Figure 4, the output-power density as well as the efficiency of the 100 and 250 nm devices behave differently depending on the illuminance. While for higher illuminances the 100 nm sample performs better, at low illuminance, the thicker device excels. The reason for this is the different significance of series and parallel resistances, since the current densities flowing through the device are considerably smaller for low light intensities compared to 1 sun operation.

#### Impact of Resistive Elements on Intensity-Dependent Device Performance

In a typical solar cell at 1 sun illumination, the series resistance *R*_s_ is an important parameter that leads to efficiency losses. In organic solar cells with their relatively low mobility as compared to other photovoltaic technologies, the nonlinear resistance of the active layers is of particular concern and can lead to losses in fill factor or short-circuit current density and inhibits the fabrication of efficient solar cells with thicker active layers.^18,29^ If organic solar cells are used for indoor applications, however, the current densities *J* flowing under normal operating conditions are substantially reduced (100 to 1000 times), which will mitigate the voltage losses Δ*V* = *JR*_s_ over series resistances in the device. However, the reduced operating voltage at lower light intensity will naturally amplify the importance of parallel resistances as well as features of the *J*–*V* curve that resemble a parallel resistance in their effect on the current voltage curve. Therefore, the description of light intensity-dependent operation of solar cells (organic or inorganic) has frequently been discussed using equivalent-circuit models that include resistive elements (series and shunt resistance).^25,30−32^ This is true even for organic photovoltaics, where equivalent-circuit models based on the principle of superposition^33,34^ have been only moderately useful^35^ to describe 1 sun operation.

Especially in organic solar cells, the shunt und series resistance is strongly affected by the thickness of the active layer. Typically, small active-layer thicknesses result in lower series and parallel resistances, although the parallel resistance underlies a certain random distribution due to possible defects in the active layer. As a result, sample series with different thicknesses are a powerful tool to examine the low light performance of solar cells.

**Figure 5:**
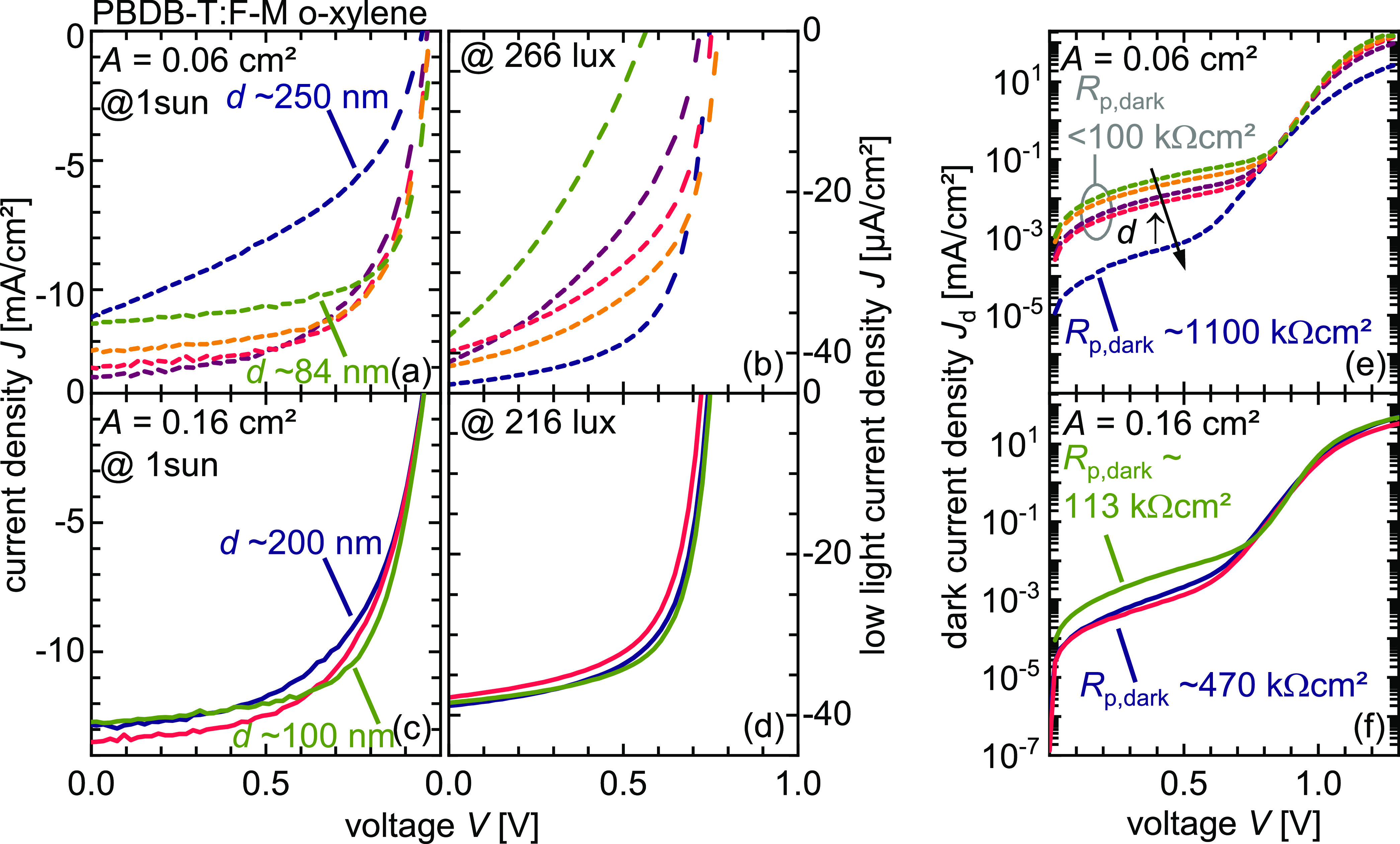
*J*–*V* curves of PBDB-T:F-M organic solar cells for different thicknesses and active areas under 1 sun (a, c), low light (b, d) conditions, and in the dark (e, f). Dashed lines show devices with an active area of *A* = 0.06 cm^2^ (a, b, e) and solid lines represent samples with an area of *A* = 0.16 cm^2^ (c, d, f). Same colors indicate samples with similar spin-coating parameters.

Figure 5a–d shows the current density–voltage (*J*–*V*) curves under 1 sun and ∼265 lux and Figure 5e,f shows the *J*–*V* curves in the dark for PBDB-T:F-M organic solar cells of different thicknesses and cell areas of 0.06 and 0.16 cm^2^. Note, that the devices with smaller active areas are measured in sample boxes with silver lids, whereas the samples with a larger active area are measured in sample boxes with a black lid. As the current of the LED is kept constant, the illuminances differ by about 23% as discussed in the Supporting Information (see Figure S3 and Note 1). The 1 sun performance parameters are plotted against the thickness in Figure S4, and the performance parameters in the low light regime are listed in Table S2.

For the 0.06 cm^2^ devices, thin solar cells under 1 sun illumination show lower photocurrents, but high fill factors of ∼70% prove efficient extraction of carriers toward the electrodes. The maximum *J*_sc_ is reached at thicknesses ∼150 nm. Thicker devices show FF < 55% as a result of extraction losses. The maximum *V*_oc_ is 0.97 V for the thinnest sample and *V*_oc_ is decreasing slightly (∼30 mV) with increasing thickness. The highest efficiency under 1 sun illumination of 8.5% is reached with the 100 nm device. Devices with *A* = 0.16 cm^2^ show similar thickness trends but achieve slightly lower *V*_oc_ and FF values at 1 sun (see Figure S4). Due to the high *V*_oc_, the high band gap material system PBDB-T:F-M is likely to perform well under low light conditions. In contrast to 1 sun illumination, the thin 0.06 cm^2^ devices show severe extraction losses (lower photocurrents, FF and *V*_oc_) under low light illumination. Samples with thicker active layers exhibit higher photocurrents (Figure 5b), which is in good agreement with increasing parallel resistances of the dark *J*–*V* curves for thicker devices (Figure 5e). Only the 100 nm device (yellow dashed line) shows a higher-than-expected low light performance while having a relatively low *R*_p,dark_. Surprisingly, the devices with a larger active area (*A* = 0.16 cm^2^) perform well at ∼216 lux independent of the active-layer thickness.

**Figure 6:**
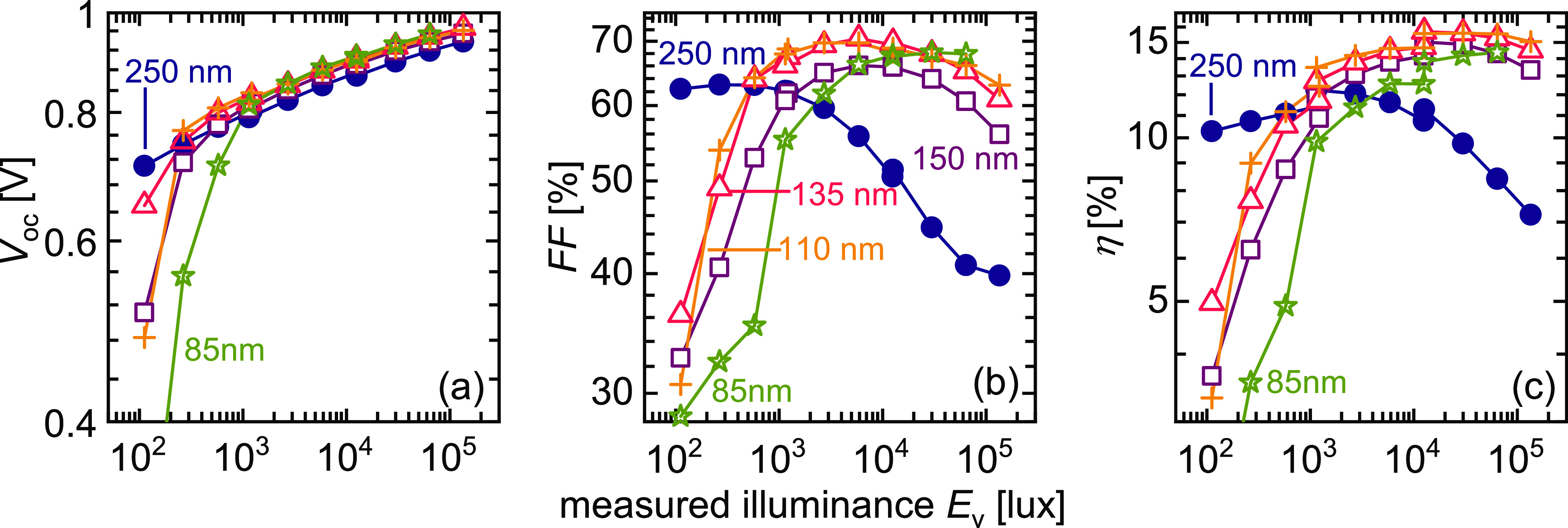
(a) Open-circuit voltage *V*_oc_, (b) fill factor FF, and (c) efficiency η of PBDB-T:F-M solar cells with *A* = 0.06 cm^2^ plotted against the illuminance on a double-logarithmic scale. Thin samples show an earlier drop of performance for decreasing illuminances. The thickest sample lacks in performance for the high intensity region but excels in the low light regime.

Now, we examine the intensity-dependent performance of the PBDB-T:F-M solar cell with *A* = 0.06 cm^2^. The *V*_oc_, the FF, and the η are depicted in Figure 6 on a double-logarithmic scale. The *J*_sc_, the *P*_out_, and the *P*_in_ are shown in Figure S5. We can classify the performance into three regions depending on the magnitude of the photocurrent flowing.^25,30^ For high intensities and photocurrents, performance is limited primarily by the voltage drop *JR*_s_ over the series resistance and collection will be hindered especially for thicker devices, resulting in low FF and η, which corresponds well with our data. The performance for intermediate intensities is linked to the diode region of the dark *J*–*V* curve and is therefore dominated by recombination.^25,30^ Here, the *V*_oc_ decreases logarithmically with decreasing illuminance as *V*_oc_ = *n*_id_*kT*/*q* ln (*J*_sc_/*J*_0_), where *n*_id_ is the ideality factor, *J*_0_ is the saturation-current density in the dark, *k* is Boltzmann’s constant, and *T* is the temperature of the solar cell. The influence of the ideality factor on the *V*_oc_ decrease will be discussed in the Determining Recombination Mechanisms with Intensity- and Thickness-Dependent Data section. The FF in this region is only slightly dependent on the light intensity, but the form of FF(*E*_v_) can be influenced by the underlying recombination mechanism.^25^ The efficiency is decreasing for decreasing light intensities and is mainly affected by the *V*_oc_ decrease as η = FF*J*_sc_*V*_oc_/*P*_in_ and *J*_sc_ ∝ *P*_in_. For the third regime with small intensities/photocurrents, the performance is dominated by the finite shunt resistance.

In this low light regime, thin devices show a drastic decrease of FF and η. The device with a 250 nm-thick active layer maintains high FF >60% and an efficiency of 10.7% for ∼200 lux. As the impact of the shunt resistance heavily depends on the voltage and the photocurrent density of a solar cell, it makes sense to put the actually measured shunt resistance into perspective. A suitable way of doing this is to define a critical shunt resistance *R*_p,crit_ = FF *V*_oc_/*J*_sc_ ≈ *V*_oc_/*J*_sc_ (see Note 2 in the Supporting Information) using the most easily available quantities for photovoltage and photocurrent.^36^ One can imagine that the slope defined by *R*_p,crit_ is the line connecting *J*_sc_ and *V*_oc_, which will always define a situation with a tremendous negative impact on device performance at a given light intensity if *R*_p,dark_ < *R*_p,crit_. As *J*_sc_ ∝ Φ and *V*_oc_ ∝ ln (Φ) + const, *R*_p,crit_ is decreasing with increasing light intensity. Devices with thin active layers, usually having lower *R*_p,dark_, reach *R*_p,crit_ for higher intensities, resulting in an early linear drop of *V*_oc_ with decreasing intensity. For the device with a thick active layer of 250 nm, the *R*_p,dark_ is sufficiently high to maintain a high *V*_oc_ even for low illuminances of 100 lux.

**Figure 7:**
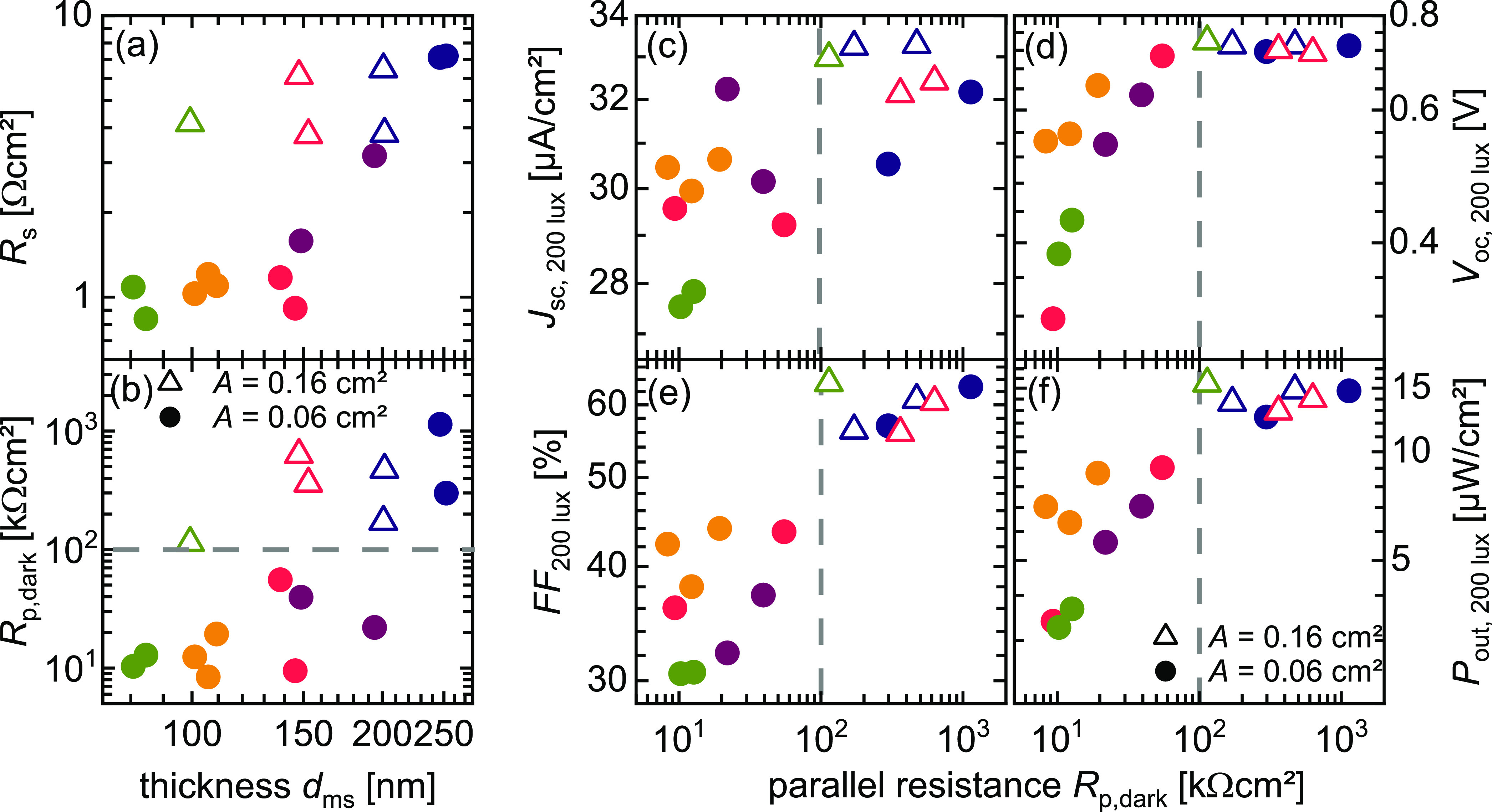
(a) Series resistance *R*_s_ and (b) parallel resistance *R*_p,dark_ plotted against the active-layer thickness *d*_ms_ on a double-logarithmic scale. Performance parameters (c) *J*_sc_, (d) *V*_oc_, (e) FF, and (f) *P*_out_ interpolated to an illuminance of 200 lux versus the parallel resistance in the dark *R*_p,dark_. The *V*_oc_ is increasing with increasing *R*_p,dark_ and saturates for *R*_p,dark_ > 100 kΩ cm^2^.

As we now have information on the intensity dependence, we are able to interpolate the performance to any illuminance, e.g., 200 lux. Consequently, a fair comparison between data sets with slightly different measuring conditions can be made (given that the LED spectrum is constant). In the following, the low light performance at 200 lux of ∼20 devices with different thicknesses and active areas are analyzed regarding the effect of parasitic resistances, which is depicted in Figure 7. Performance parameters under 1 sun condition are plotted against *R*_p,dark_ in Figure S6.

In Figure 7a,b, the *R*_s_ and the *R*_p,dark_ are depicted as a function of the active-layer thickness, respectively. While samples with an active layer of *A* = 0.06 cm^2^ show a steep increase of *R*_p,dark_ with increasing thickness, the devices with *A* = 0.16 cm^2^ exhibit overall higher *R*_p,dark_ with a less pronounced increase. Although the effect of different active areas can be significant,^37^ a direct comparison of devices with different active areas is not the focus of this study. Nevertheless, our data reveals that dark shunt resistances can scale differently with thickness depending on the active area and therefore affect device performance, especially in the low light regime. In Figure 7c,d, the *J*_sc_ and the *V*_oc_ at 200 lux are plotted against the *R*_p,dark_. Due to the short-circuit condition, the *J*_sc_ is not dependent on *R*_p,dark_, but rather on the active-layer thickness (compare Figure S4). The *V*_oc_ is increasing with increasing *R*_p,dark_ and saturates for *R*_p,dark_ > 100 kΩ cm^2^ around *V*_oc_∼0.7 V, which is only ∼250 mV lower compared to the *V*_oc_ at 1 sun. The FF also reaches a plateau for *R*_p,dark_ > 100 kΩ cm^2^, but the dependence is more scattered. With the concept of determining the low light performance by means of *R*_p,dark_, it is not possible to explain why the FF still scatters around ±10% for a constant *R*_p,dark_. The *P*_out_ increases for increasing *R*_p,dark_ and displays a plateau of maximum values for *R*_p,dark_ > 100 kΩ cm^2^ about ∼15 μW/cm^2^. The values below this threshold show noticeably more scattering compared to the plateau, which is mainly a consequence of the scattered FF in this region. For the devices with *A =* 0.06 cm^2^, the maximal *P*_out_ of 14.7 μW/cm^2^ is reached for the 250 nm active-layer thickness, resulting in a *J*_sc_ of 32 μA/cm^2^, a *V*_oc_ of 0.73 V, and an FF of 62.7%. As the devices with an *A* = 0.16 cm^2^ have overall *R*_p,dark_ > 100 kΩ cm^2^, the maximal *P*_out_ is not a function of the active-layer thickness and the best *P*_out_ is 15.5 μW/cm^2^ (*J*_sc_ = 33 μA/cm^2^, *V*_oc_ = 0.74 V, FF = 63.4%) for the 100 nm device.

**Figure 8:**
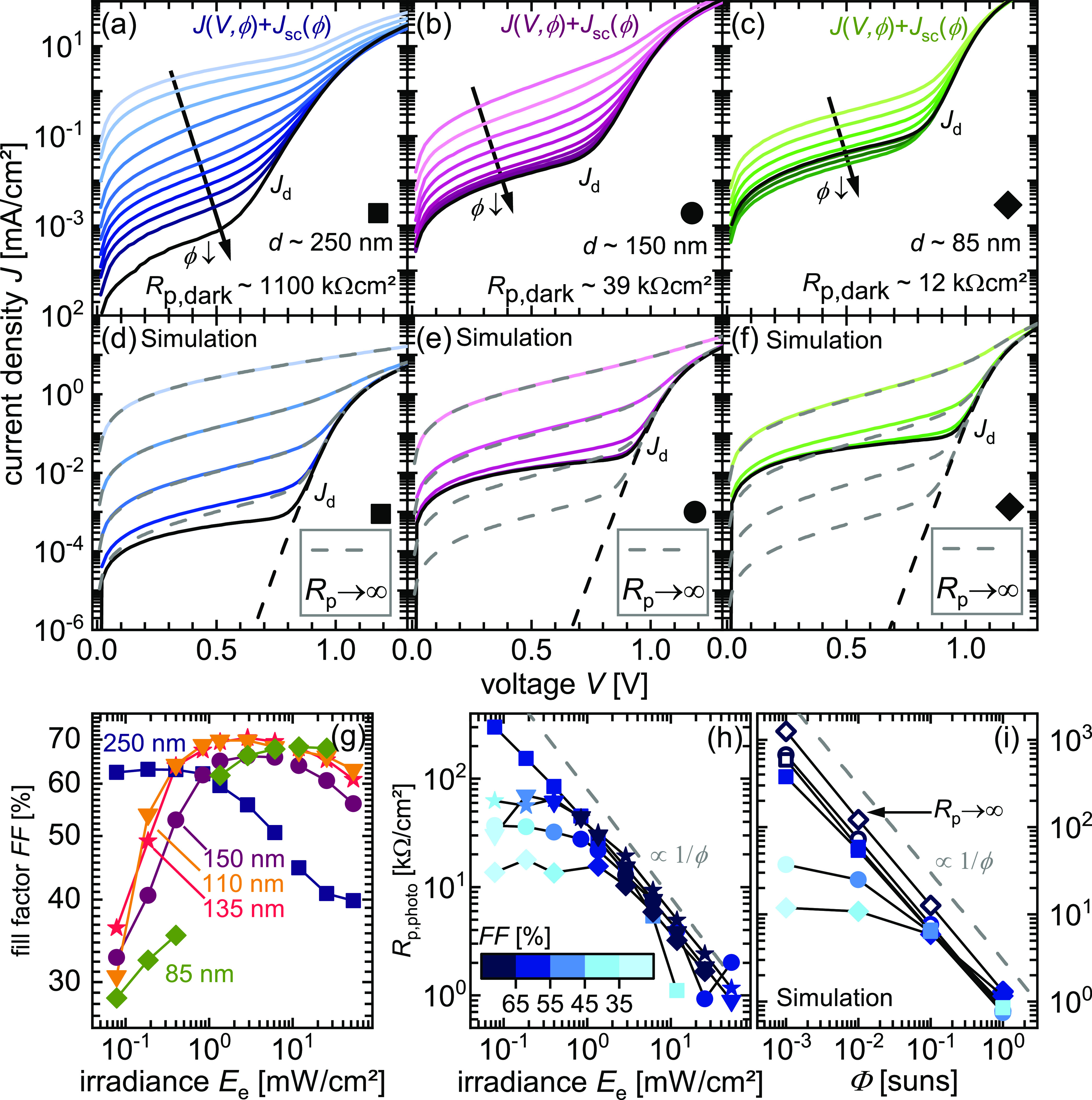
Measured (a–c) and simulated (d–f) shifted illuminated *J*–*V* curves *J*(*V*, Φ) + *J*_sc_(Φ) (solid lines) and *J*–*V* curves in the dark (black solid lines) for devices with active-layer thicknesses of 85 (a, d), 150 (b, e), and 250 nm (c, f) and *A* = 0.06 cm^2^. In (d–f), dashed lines represent the case for infinite shunt resistance. (g) Corresponding FF plotted against the irradiance. (h, i) Photo-shunt resistance of the measured (h) and simulated (i) shifted *J*–*V* characteristics versus the irradiance for devices with different thicknesses. Dark blue symbols correspond to high FF, and light blue symbols correspond to low FF. As long as the photo-shunt *R*_p,photo_ scales inversely with the irradiance, high FF are maintained.

With the concept of the shunt resistance in the dark, it is not possible to explain why the FF in the region below *R*_p,dark_ < 100 kΩ cm^2^ is more scattered, or in other words, all samples with a constant *R*_p,dark_ should result in a constant FF if a simple equivalent-circuit model was a good approximation for the *J*–*V* curves of organic solar cells. The dark *J*–*V* curve *J*_d_ takes the recombination of dark charges and losses due to shunts and pinholes into account. Hence, we also need to consider the finite efficiency of collecting photogenerated charges to understand the situation for illuminated solar cells. In a hypothetical ideal case, all generated charges are collected. The surplus of charges due to illumination contributes to the illuminated *J*–*V* curve with a collection efficiency *f*_c_ = 1, independent of the applied voltage. The voltage dependence of the illuminated *J*–*V* characteristic is then dominated by the dark *J*–*V* curve and can be described by superposition^34^*J*(*V*, Φ) = *J*_d_(*V*) – *J*_sc_(Φ). In contrast, in a realistic organic solar cell, the recombination current will depend on both voltage and light intensity, which implies that the superposition principle is no longer valid. This absence of superposition has been alternatively described in the literature by either an illumination dependence of the recombination current^38^ or a voltage dependence of the photocurrent.^39^ Both approaches only differ in terminology but not with respect to the physical effect. A simple way to distinguish between the ideal case with a voltage-independent photocurrent (*J*_sc_) and the nonideal case with a voltage-dependent photocurrent *J*_ph_ is to plot the sum *J*(*V*, Φ) + *J*_sc_(Φ) (with *J*_sc_ > 0), which is depicted in Figure 8a–c for different thicknesses and illumination intensities. This approach of shifting the light *J*–*V* curves into the first quadrant and plotting them logarithmically has been infrequently used in various photovoltaic technologies^33,34,38,40,41^ starting with Si in the late 1970s but has so far not been discussed in the context of organic photovoltaics. The shifted *J*–*V* curve resembles a dark *J*–*V* curve and represents the deviation from the voltage-independent ideal case *J*(*V*, Φ) + *J*_sc_(Φ) = *J*_d_(*V*) – (*J*_ph_(*V*, Φ) – *J*_sc_(Φ)) = *J*_d_(*V*) – Δ*J*_ph_(*V*, Φ), where *J*_ph_ > 0 and Δ*J*_ph_ < 0. Instead of analyzing total current densities, we only consider changes of the photocurrent Δ*J*_ph_ relative to the ideal case at short-circuit. The higher the shifted *J*–*V* curves, the more carriers are still present in the devices, which were not extracted. Therefore, higher shifted *J*–*V* curves will represent a situation with higher extraction losses, as the difference Δ*J*_ph_ to the ideal voltage-independent case at short-circuit is higher. For the thickest device with the 250 nm active layer, the shifted *J*–*V* curves lie above the dark *J*–*V* curve (Figure 8a), showing the photocurrent to be voltage dependent. For decreasing intensities, the shifted *J*–*V* curves decrease gradually and good charge extraction is maintained (for voltages <0.6 V, for higher voltages injection of charge carriers dominates). For thin devices and high intensities, the behavior is comparable to the characteristics of the thick device. In contrast, at low intensities, the shifted *J*–*V* curves accumulate close to or below the dark *J*–*V* curve. This situation is associated with dominant leakage currents through the shunt resistance, as the shifted *J*–*V* curves are not decreasing with decreasing light intensity anymore. Furthermore, we can compare shifted *J*–*V* curves at constant intensities but different thicknesses. For the highest intensity, thinner devices show lower shifted *J*–*V* curves, implying better charge extraction since the voltage drop *JR*_s_ is smaller for thin devices. For the lowest intensity, the thickest device will have the best charge extraction as it is the only device, which is not limited by the dark *J*–*V* curve.

In order to prove that shifted *J*–*V* curves are limited by the dark *J*–*V* curve, we performed drift-diffusion simulations with the Advanced Semiconductor Analysis^42,43^ (ASA) software of a generic organic solar cell considering bimolecular and SRH recombination for intensities from 10^–3^ to 1 sun. All parameters for the simulation (Table S3) and the *J*–*V* curves (Figure S7) can be found in the Supporting Information. In Figure 8d–f, the simulated shifted *J*–*V* curves (solid lines), resulting from calculations with the same *R*_p,dark_ as in the experiments, are depicted, showing that the experimental data is well reproduced. As the results are independent of the recombination mechanism (see Figure S7), different recombination mechanisms cannot be the origin of the accumulated shifted *J*–*V* curves for low intensities. Instead, we see that in the case of an infinite *R*_p,dark_ (dashed gray lines in Figure 8d–f), the shifted *J*–*V* curves continue to decrease for decreasing light intensity and no accumulation occurs (no losses due to dominant leakage currents).

Furthermore, we can apply the same fitting procedure according to a simple equivalent circuit to the shifted illuminated *J*–*V* curves (see Supporting Information Figure S8 and Note 3). This results in a photo-shunt resistance *R*_p,photo_, which is simply a measure of the voltage dependence of the photocurrent Δ*J*_ph_(V, Φ) at different light intensities and depicts the underlying generation and recombination events. Now, we can examine how the *R*_p,photo_ evolves with intensity, which is connected to the resulting FF. For this purpose, in Figure 8g, the FFs of the devices with *A* = 0.06 cm^2^ and, in Figure 8h,i, the experimental and simulated photo-shunt resistances *R*_p,photo_ are plotted versus the irradiance on a double-logarithmic plot, respectively. The photo-shunt resistance is inversely proportional to the light intensity for high intensities (Figure 8h,i). The shifted *J*–*V* curves are connected to the generation and recombination of charges via *J*(*V*, Φ) + *J*_sc_(Φ) = *J*_d_(*V*) – Δ*J*_ph_(*V*, Φ) and *J*_ph_(Φ, *V*) = *q* ∫ (*G*(*x*, Φ, *V*) – *R*(*x*, Φ, *V*)) d*x*). Therefore the *R*_p,photo_ ∝ Φ^–1^dependence can be explained by the linear increase of *J*_ph_ with intensity (at a constant voltage). In the region for *R*_p,photo_ ∝ Φ^–1^, good *FF*s are maintained in experiment and simulation. In regions where shifted *J*–*V* curves accumulate close to the dark *J*–*V* curve, we see constant plateaus of *R*_p,photo_, which are associated with low FFs. Therefore, the *R*_p,photo_ is limited by the *R*_p,dark_, which illustrates the importance of the dark-shunt resistance in the low light regime.

Interestingly, the region of constant *R*_p,photo_ does not only indicate low FFs but also the *R*_p,photo_ correlates with the FF (the lower *R*_p,photo_, the lower the FF). While the sole consideration of the dark-shunt resistance *R*_p,dark_ cannot explain the scattered FFs at a constant *R*_p,dark_ (see region of low *R*_p,dark_ in Figure 7e), a low *R*_p,photo_ is a surprisingly sensitive measure of low FF (for more samples see Supporting Information Figure S9). Overall, the simulations match well with the experimental data and prove that solar cell performance in the low light regime is limited by the *R*_p,dark_. Note that the experimental shifted *J*–*V* curves can lie below the dark *J*–*V* curve for small intensities, which is associated with a positive Δ*J*_ph_ > 0 and |*J*_ph_ | > | *J*_sc_| (see Supporting Information Figure S10). In general, we would expect |*J*_ph_ | < | *J*_sc_| as the photocurrent at a certain voltage has higher extraction losses compared to the short-circuit condition. A more detailed discussion can be found in the Supporting Information Note 4. Nevertheless, the introduction of shifted *J*–*V* curves and the concept of the photo-shunt resistance can explain regions of low *FF* suffering from extraction losses and is therefore a powerful tool to evaluate and explain the low light performance of solar cells.

**Figure 9:**
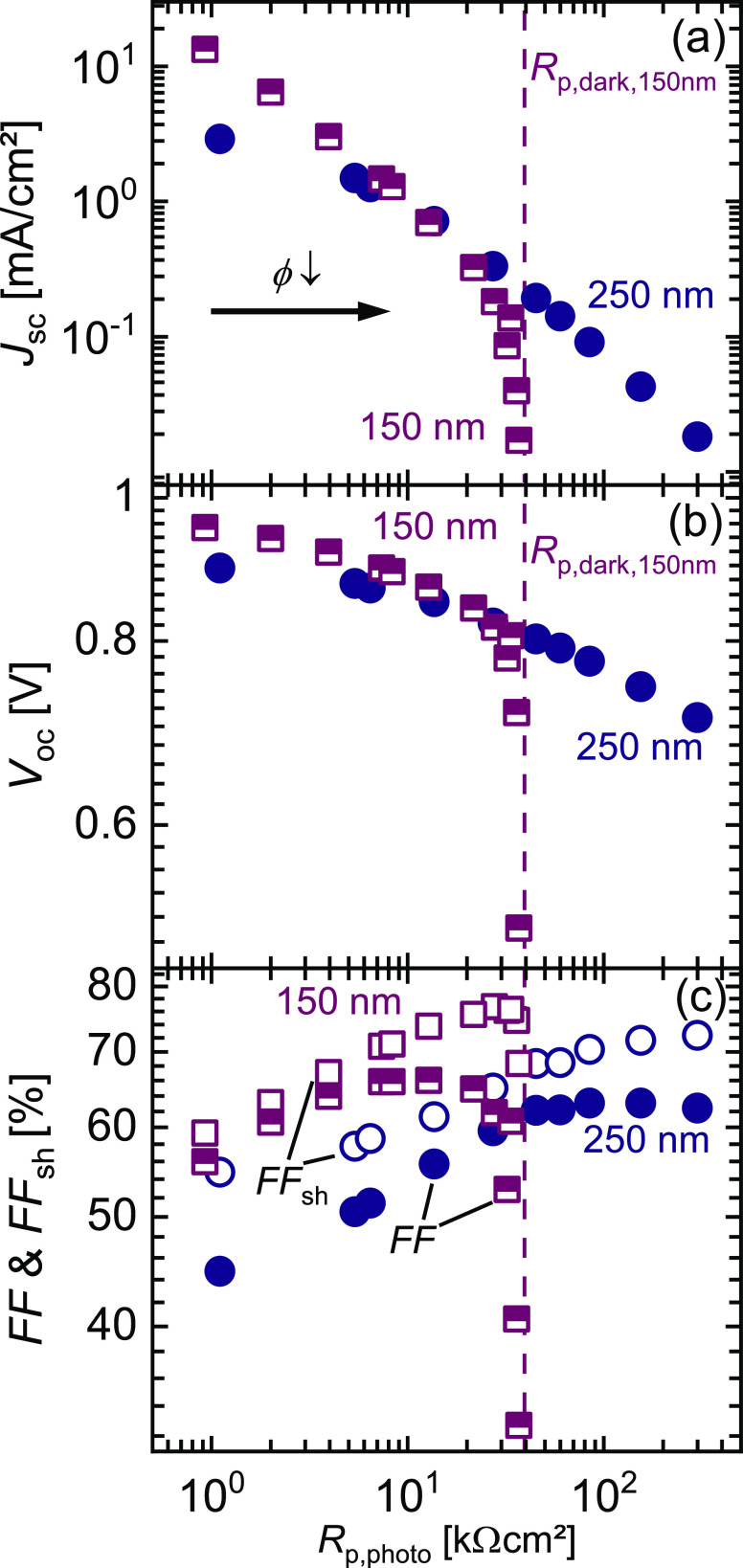
(a) Short-circuit current density *J*_sc_, (b) open-circuit voltage *V*_oc_, (c) fill factor FF, and calculated fill factor FF_sh_ (open symbols) plotted against the photo-shunt resistance *R*_p,photo_ on a double-logarithmic scale for the devices with thicknesses of 150 (squares) and 250 nm (spheres) and *A* = 0.06 cm^2^. The dashed line indicates the dark-shunt resistance *R*_p,dark_ of the 150 nm sample. The *R*_p,dark_ of the 250 nm sample is 1100 kΩ cm^2^. For more samples, see Supporting Information Figure S11.

In this section, we analyze the influence of the photo-shunt resistance on all performance parameters exemplary for a thin and the thickest solar cell. In Figure 9, the *J*_sc_ (a), *V*_oc_ (b), and FF (c) are plotted against the *R*_p,photo_. For regions where *R*_p,photo_ ∝ 1/ϕ samples show good performance and for regions where the *R*_p,photo_ saturates with the light intensity, the performance of devices breaks down. The beginning of the steep decrease coincides vaguely with the beginning of *R*_p,photo_ ≈ *R*_p,dark_. As the light intensity and thus photocurrents are small, higher shunt-resistances would be needed to maintain good performance (*R*_p,crit_ ∝ 1/*J*_sc_). For the thick solar cell (blue spheres), the *R*_p,dark_ is not reached and good performance is preserved throughout the whole intensity range. The *V*_oc_ shows a less pronounced decrease with increasing *R*_p,photo_ compared to the *J*_sc_ as the *V*_oc_ depends logarithmically on light intensity. The FF shows an increase with increasing *R*_p,photo_ for both devices (150 and 250 nm) as a consequence of a less pronounced influence of the series resistance with decreasing light intensity (less current density). In addition to the measured FF, the FF_sh_ according to the empirical equation for fill factors by Green is calculated (for devices suffering from shunt and series resistances losses, see Supporting Information Note 5).^44^ As can be seen in Figure 9c both FF_sh_(*R*_p,photo_) trends can only be vaguely described by the empirical approach. In general, for higher intensities the empirical equation overestimates the FF about 2–20% and the deviation increases for increasing intensities. In the region of low light intensities (<1000 lux), the overestimation gets worse and can reach 100%.

In conclusion, we proposed a method to identify extraction losses with *J*–*V* measurements in the low light regime. If the shifted *J*–*V* curves increase gradually with light intensity, the introduced photo-shunt resistance is inversely proportional to the light intensity and good FFs are maintained. On the other hand, accumulated shifted *J*–*V* curves cannot fall considerably below the dark *J*–*V* curve, which leads to constant plateaus of the photo-shunt resistance. The limitation of the photo-shunt to the dark-shunt resistance is associated with extraction losses and low FF. Furthermore, the performance of solar cells with lower shifted *J*–*V* curves (or higher photo-shunt resistances) is superior, if compared to other solar cells at the same light intensity.

#### Determining Recombination Mechanisms with Intensity- and Thickness-Dependent Data

In the mid-intensity range (once a sufficient shunt resistance is ensured), device performance is limited by the *V*_oc_ decrease with light intensity. For higher ideality factors, the *V*_oc_ decreases faster with decreasing light intensity (as the slope of d*V*_oc_/dln(Φ) ∝ *n*_id_). Thus, higher ideality factors have a tremendous negative impact on performance in the mid-intensity regime. One way to probe recombination dynamics is to examine intensity-dependent data sets as the dominant recombination mechanism usually changes with intensity. The evaluation of ideality factors can be used to determine the dominant recombination mechanism. The recombination of a free hole and a free electron scales as *R*(*x*) ∝ *np* ∝ exp (Δ*E*_f_/*kT*), where *R*(*x*) is the recombination rate at position *x*, *n*, and *p* are the carrier concentrations of free electrons and holes, Δ*E*_f_ is the quasi-Fermi level splitting, and *kT* is the thermal voltage. Thus, for bimolecular recombination of free charges, the ideality factor is 1. If recombination of, for example, a free hole with a trapped electron in a midgap state is dominant (assuming an intrinsic semiconductor), then *R*(*x*) ∝ *p* ∝ exp (Δ*E*_f_/2*kT*) and the ideality factor is 2. Recombination with trap states other than midgap leads to ideality factors between 1 and 2, and the closer the ideality factor is to 2, the more midgap states are involved in recombination.^45^ One way to probe the recombination of charge carriers is to calculate the inverse slope of the logarithmic dark current density versus the voltage differentially.
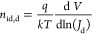
6

As the dark *J*–*V* curve is influenced by the series and shunt resistance, it is not clear if changes in *n*_id,d_ will be due to recombination or due to changes caused by the parasitic resistances. Another way to determine the ideality factor is to measure the voltage at open circuit at different light intensities:
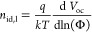
7

In our particular case where we used different ND filters with slightly different transmission spectra, we need to replace the light intensity Φ with the *J*_sc_. The *V*_oc_(Φ) data lead to jumps where we changed filters, while the *V*_oc_(*J*_sc_) is not affected by the filter change.

**Figure 10:**
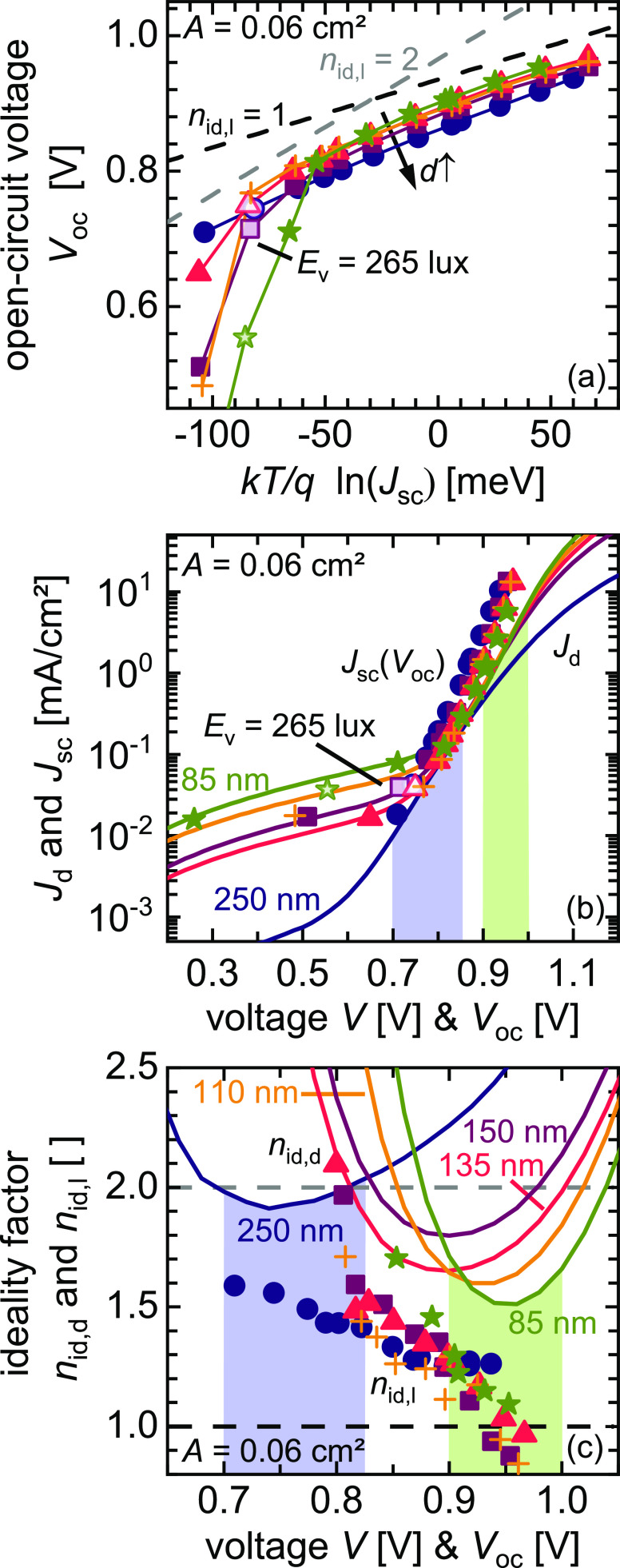
(a) *V*_oc_ plotted against *kT*/*q* ln (*J*_sc_) for solar cells with different thicknesses and *A* = 0.06 cm^2^. The black and gray dashed lines indicate slopes with *n*_id_ = 1 and *n*_id_ = 2, respectively. The transparent symbols show the measurement corresponding to 265 lux. (b) Dark *J*–*V* curve and *J*_sc_(*V*_oc_) data plotted into one graph. While the dark *J*–*V* curves show influence of both parasitic resistances, the *J*_sc_(*V*_oc_) is only affected by the shunt resistance. The light blue and green areas mark the exponential regions of the 250 and 85 nm-thick solar cells as a guide to the eye. (c) Dark and light ideality factor *n*_id,d_ and *n*_id,l_ as a function of voltage. While *n*_id,d_ is affected heavily by the series and the shunt resistance, the *n*_id,l_ only shows influence of the shunt resistance at low voltages.

In Figure 10a, the *V*_oc_ is plotted versus *kT*/*q* ln (*J*_sc_) for solar cells with different thicknesses, resulting in a plot where the slope is proportional to the light ideality factor. The black and the gray dashed lines indicate slopes corresponding to an ideality factor of 1 and 2, respectively. While at low intensities of 265 lux (indicated by transparent symbols) the solar cell is dominated by the shunt resistance (*n*_id,l_ > 2), we see a gradual decrease from higher ideality factors to lower ideality factors with increasing light intensity. The decrease in *n*_id,l_ is resulting from a transition between two recombination mechanisms. In order to identify the regions that are affected by parasitic resistances, in Figure 10b, the dark *J*–*V* curves and the *J*_sc_(*V*_oc_) data are plotted into one graph. The dark *J*–*V* curve is affected by both parasitic resistances for low and high voltages, resulting in small exponential regimes for average voltages (marked exemplarily for 85 and the 250 nm sample with light green and blue rectangles). If the dark *J*–*V* curve is interpreted in terms of the dark ideality factor *n*_id,d_ (eq 6), only this exponential regime can be considered. The *J*_sc_(*V*_oc_) pairs of thinner samples (<250 nm) show a substantial shunt influence in the low voltage regime. The evaluation of the light ideality factor *n*_id,l_ in this region will lead to distorted, higher apparent values. The *J*_sc_(*V*_oc_) of the thickest solar cell (250 nm) is hardly affected by the shunt resistance. For higher *V*_oc_, the *J*_sc_ is exponentially increasing with increasing *V*_oc_ and is not following the series resistance kink compared to the dark *J*–*V* curve.

In Figure 10c, the light and dark ideality factors are depicted versus the voltage/*V*_oc_. In contrast to a simple fit of the dark *J*–*V* curve (or alternatively a fit to the *V*_oc_(*J*_sc_) data in Figure 10a), the differential determination of the ideality factor according to eqs 6 and 7 include information about the voltage-dependence and thus about the influence of the *R*_s_ and *R*_sh_.^45^ As the *n*_id,d_ is proportional to the inverse slope of ln(*J*_d_), we see a dramatic increase where the *R*_s_ and *R*_sh_ have a substantial influence. Compared to the thin samples, the exponential region of the *J*–*V* curve of the 250 nm sample is at lower voltages (marked with the light blue rectangle), resulting in a left-shifted parabola. The minimum of the 85 nm sample is at 0.95 V with an *n*_id,d_ = 1.5 and the samples with a 250 nm active layer shows a minimum at 0.75 V and *n*_id,d_ = 1.9. This difference in the dark ideality factor can be caused by superimposed resistive effects, as it is not clear if the value of the ideality factor already saturated to the value it would have without resistive elements being present. Hence, the shift of the minimum of *n*_id,d_ is most likely resulting from the influence of the parasitic resistances and is not due to any change in recombination mechanism.^45^ The *n*_id,l_ data allows a more precise interpretation of the recombination mechanism as it is not affected by the series resistance. The *n*_id,l_ is slightly lower than *n*_id,d_ and is linearly decreasing with increasing *V*_oc_. For samples with an active-layer thickness of <250 nm, the *n*_id,l_ is dramatically increasing at lower voltages (intensities). In this region, the *V*_oc_ is decreased due to the influence of low shunt resistances, leading to higher apparent ideality factors.^31^ Thus, it is of great importance to identify regions with dominant leakage currents to prevent misinterpretations regarding the ideality factor. In the region not affected by the shunt, the *n*_id,l_ of all samples overlap, suggesting no influence of the active-layer thickness on the recombination mechanism at a constant voltage. The linear decrease of *n*_id,l_ with *V*_oc_ indicates a transition between two recombination mechanisms. For lower *V*_oc_, the *n*_id,l_ > 1 implies trap-assisted recombination, which has negative impact on device performance as discussed above. At voltages of ∼0.95 V, *n*_id,l_ is decreasing to *n*_id,l_ ≈ 1, indicating recombination of free charges. Above 0.95 V, *n*_id,l_ < 1, which was interpreted in terms of surface recombination.^45^ In conclusion, the differential calculation of the light ideality factor shows that performance is limited by dominant SRH recombination in the mid-intensity regime.

In the scope of analyzing performance of solar cells, it is important to know whether the recombination predominantly occurs in the bulk material or via recombination at the surface. If direct recombination of free charges dominates in the bulk, the recombination current scales linearly with the thickness *d* of the active layer.^46^ On the other hand, if recombination is governed by surface recombination and the surface recombination velocity is low, charges diffuse to the wrong electrode and the recombination current is not a function of the active-layer thickness. In the case of a high surface recombination velocity, the transport of charge carriers is the limiting factor and charges will reach the electrode later for thicker devices. Hence, the recombination current is decreasing for increasing active-layer thicknesses. Thus, we can distinguish between bulk and surface recombination if we measure the recombination current of a set of devices with different active-layer thicknesses and observe how the recombination current scales with the thickness. One way to probe the recombination current *J*_rec_ is to consider an illuminated solar cell at open-circuit conditions, where recombination equals generation (*J*_gen_ = *J*_rec_) and no net current is flowing. Derived from the simple diode equation, one can then define a saturation-current density^46^
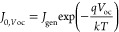
8

Here, *J*_gen_ is the generated photocurrent and can be estimated by the photocurrent *J*_photo_ = *J*_light_ – *J*_dark_ at reverse bias, where the collection of charges is efficient and recombination is negligible. The dependence of *J*_0,*V*oc_ on *d* can be examined by plotting *J*_0,*V*oc_ and *d* on a double-logarithmic axis and by then calculating the slope *m* = d ln (*J*_0,*V*oc_)/d ln (*d*). Zonno et al. showed for various organic solar cells under 1 sun irradiation that *J*_0,*V*oc_ ∝ *d* in the case of direct recombination in the bulk material (*m* = 1) and *J*_0,*V*oc_ ≠ *f*(*d*) for dominant surface recombination (*m* = 0, low surface recombination velocities).^46^ Although Zonno et al.^46^ illustrated the effect of direct and SRH recombination on the *J*_0,*V*oc_ in their simulations, the focus of the study is on the discrimination between surface and bulk recombination. Zonno et al.^46^ observed in their simulation that while *J*_0,*V*oc_ ∝ *d* for direct recombination (*m* = 1), the *J*_0,*V*oc_ scaled super linearly with the active-layer thickness for SRH recombination (*m* > 1) at 1 sun irradiation. When we perform intensity-dependent measurements instead of measurements at 1 sun, we change the dominant recombination mechanism, as the recombination rate for SRH recombination scales with the charge-carrier concentrations as *R*_SRH_ ∝ *n* and for direct recombination with *R*_dir_ ∝ *n*^2^. As the recombination current probed by *J*_0,*V*oc_ is directly influenced by the underlying recombination, we can not only discriminate between surface and bulk recombination but also investigate the recombination mechanism in the linear regime of *J*_0,*V*oc_ (on the double-logarithmic scale). Here, we extend the study of Zonno et al.^46^ to our intensity-dependent data set and elaborate on the effect of different recombination mechanisms on the linear regime.

**Figure 11:**
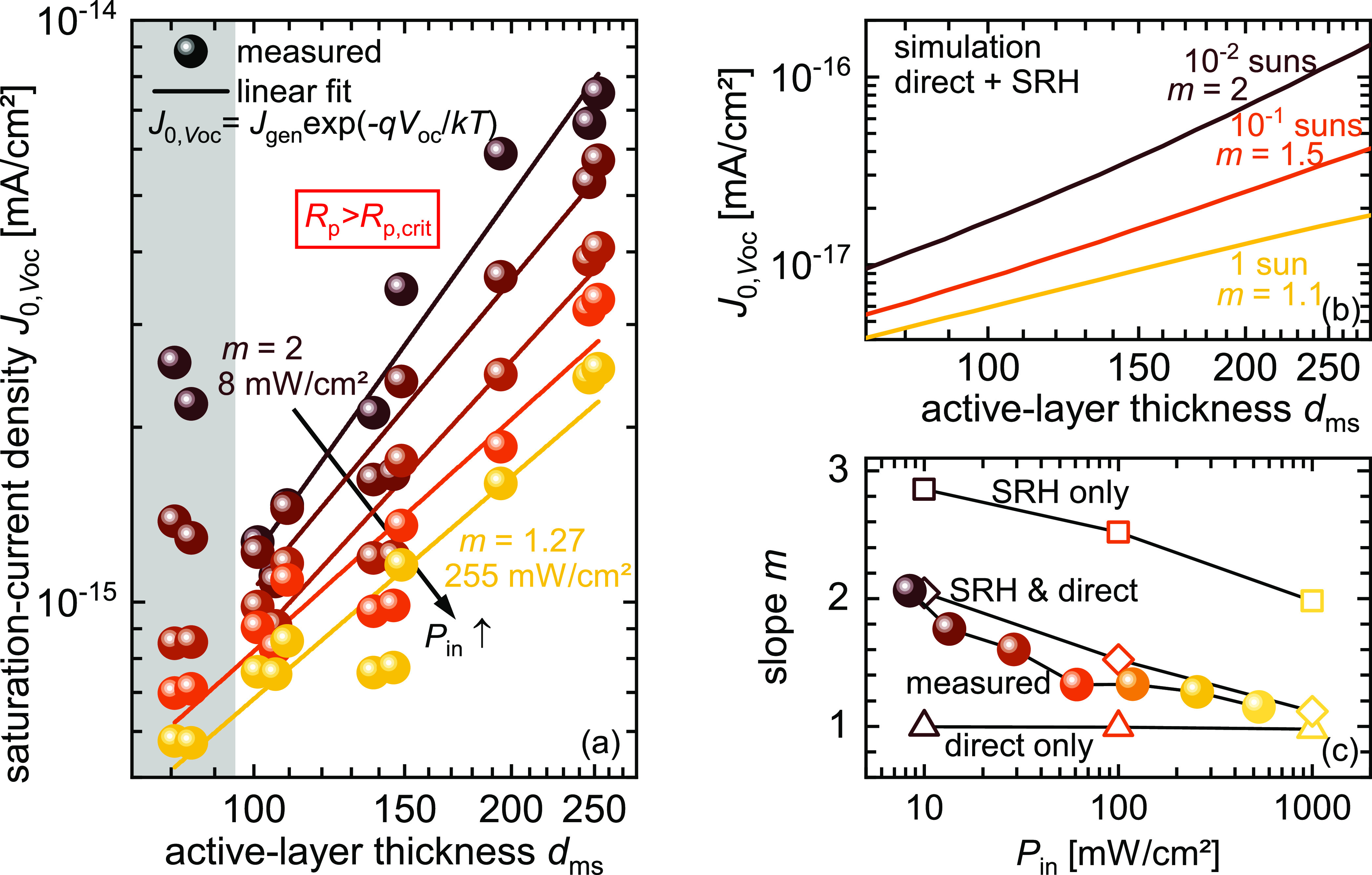
(a) Measured saturation-current density *J*_0,*V*oc_ according to eq 8 plotted against the active-layer thickness on a double-logarithmic scale for devices with an active area of *A* = 0.06 cm^2^ and for different light intensities (8, 13, 29, 61, and 250 mW/cm^2^). Only data in the intensity regime was used where *V*_oc_ is not affected by leakage currents through the *R*_p_ and *V*_oc_ ∝ ln (Φ) + const holds true. Straight lines are fits to the data. (b) Simulated *J*_0,*V*oc_ plotted against the active-layer thickness on a double-logarithmic scale. (c) Slope *m* = d ln (*J*_0,*V*oc_)/d ln (*d*) for measured data (spheres), for simulated data considering only SRH (squares) and only direct recombination (triangles) and for simulated data considering SRH and direct recombination (diamonds).

In Figure 11a, the calculated saturation-current density *J*_0,*V*oc_ is plotted against the active-layer thickness for intensities >8 mW/cm^2^ (∼1200 lux) for devices with *A* = 0.06 cm^2^. Note that the *V*_oc_ in the low-intensity regime (< 8 mW/cm^2^) can be reduced by leakage currents through the *R*_p_ (see Figure 6a and Figure S12), which can lead to higher apparent *J*_0,*V*oc_.

For active-layer thicknesses of >100 nm, the *J*_0,*V*oc_ is increasing with increasing thickness, which can be attributed to bulk recombination. For thin active-layer thicknesses (<100 nm, gray highlighted area), the *J*_0,*V*oc_ is decreasing or constant with increasing absorber thicknesses, which might suggest dominant surface recombination.^46^ Additionally, we see a splitting of data points originating from different intensities, namely, for smaller intensities, higher values of *J*_0,*V*oc_ are reached. Higher *J*_0,*V*oc_ for lower intensities is caused by the increasing importance of SRH recombination at lower intensities and consequently lower values of *V*_oc_ (see Figure S13). Furthermore, the slope *m* in the linear regime (on the double-logarithmic axis of Figure 11a), is decreasing with increasing intensities. In order to disentangle the effects of direct and SRH recombination, we performed intensity-dependent drift-diffusion simulations for three different cases (see Table S3), namely, for only direct recombination, only SRH recombination, and for a combination of both mechanisms. The simulated *J*_0,*V*oc_s of direct only and SRH only recombination are displayed in Figure S14a–c. In Figure 11b, the simulated *J*_0,*V*oc_s for the case of mixed recombination for the intensities comparable to the intensities in our experiments are shown. The intensity-dependent splitting as well as the slopes *m*, which are depicted in Figure 11c, are in good agreement with our experimental data. Note that the simulations show around two orders of magnitude higher *J*_0,*V*oc_s, since the simulations reached slightly better *V*_oc_ compared to our experiments (around 100 mV).

If only direct recombination is considered, we see no splitting of *J*_0,*V*oc_ with intensity and the slope *m* = d ln (*J*_0,*V*oc_)/d ln (*d*) is constantly 1 (Figure S14c). For all cases with *n*_id,l_ > 1 (mixed and SRH recombination), *m* increases to values above 1 for decreasing light intensities. We found an analytical approach to describe the slope *m*, which is derived in the Supporting Information Note 6. This derivation is based on the observation that the open-circuit voltage in simulation and experiment decreases linearly with the logarithm of the thickness. Then, the *J*_0,*V*oc_ scales with the active-layer thickness as
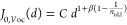
9where, *C* is a constant and β is the positive linear slope β = d*V*_oc_/d ln (*d*) if *V*_oc_ is plotted against ln(*d*) (see Figure S14g–i). Hence, the slope *m* is determined by the thickness dependence of *V*_oc_ and ideality factor *n*_id,l_, which are both a function of light intensity (see Figure S14), and consequently, the slope *m* decreases for increasing light intensities. Here, we see that not only a lower *n*_id,l_ but also a smaller decrease of *V*_oc_ with thickness would lead to a situation with increased direct recombination, which would be accompanied by an increase of the solar cells’ performance.

## Conclusions

We examined how different loss mechanisms affect the performance of PBDB-T:F-M organic solar cells at different light intensities and how to quantify and identify these loss mechanisms. These mechanisms include losses due to insufficient shunt resistances in the low light regime and losses caused by the *V*_oc_ decrease in the mid-intensity regime. First, we carefully defined our measuring conditions by assessing spectral irradiance data of our used LED with several ND filters. Then, we examined the influence of the shunt resistance (by changing the active-layer thickness) on device performance and found that a minimum shunt of ∼100 kΩ cm^2^ is sufficient for intensities ∼200 lux. Furthermore, we introduced the concept of the photo-shunt resistance, which is a consequence of the existence of different charge-carrier extraction losses at different light intensities. We found that performance is maintained as long as the photo-shunt resistance scales inversely with intensity and breaks down for constant photo-shunt resistances. Our findings were supported by drift-diffusion simulations, which indicate that the extraction losses in the low light intensity regime are limited by the dark-shunt resistance. We further examined losses in the mid-intensity regime, which are connected to recombination dynamics via the open-circuit voltage and the ideality factor. The determination of ideality factors showed that with increasing light intensity, the dominant recombination mechanism is changing from SRH to direct recombination. Furthermore, we identified dominant bulk recombination for active-layer thicknesses of >100 nm by studying the thickness-dependent saturation-current density at open circuit. We extended a method introduced by Zonno et al.^46^ to our intensity-dependent data and verified with drift-diffusion simulations that the slope d ln (*J*_0,*V*oc_)/d ln (*d*) depends on the thickness-dependence of the open-circuit voltage and on the ideality factor. This study reveals thickness- and intensity-dependent data to be a valuable tool to gain insights into performance limiting mechanisms of organic solar cells in the low light regime.

## Methods

### Device Fabrication

A structured indium tin oxide (ITO) layer on glass is treated in distilled water, acetone, and isopropyl alcohol in an ultrasonic cleaner for 10 min. Then, samples are dried on a hot plate for 10 min at 100 ° C, followed by a treatment with an oxygen plasma. For the electron transport layer, we used zinc oxide (ZnO) with a sol–gel process. Zinc acetate dihydrate (Sigma Aldrich, 100 mg), 2-methoxyethanol (Alfa Aesar, 1.5 mL), and ethanol amine (Sigma Aldrich, 28 μL) are stirred at 60 °C overnight. Prior to processing, the solution is filtered (0.45 μm PVDF filter). On top of the cleaned ITO substrate, the sol–gel is spin-coated in air with 6000 rpm for 50 s for the 0.06 cm^2^ devices and with 7000 rpm and 50 s for the 0.16 cm^2^ devices followed by an annealing step at 200 °C for 20 min. The ZnO layer is approximately ∼30 nm thick. For the active-layer, spin coating samples are transferred into a glovebox.

The polymer PBDB-T poly[[4,8-bis[5-(2-ethylhexyl)-2-thienyl]benzo[1,2-b:4,5-b′]dithiophene-2,6-diyl]-2,5-thiophenediyl[5,7-bis(2-ethylhexyl)-4,8-dioxo-4H,8H-benzo[1,2-c:4,5-c′]dithiophene-1,3-diyl]] and the NFA F-M 4,4,7,7,12,12-octyl-7,12-dihydro-bis[ethylidyne(3-oxo-methyl-1H-indene-2,1(3H)-diylidene)]]bis-4H-thieno[2″,3″:1′,2′] indeno[5′,6′:5,6]-*s*-indaceno[1,2-*b*]thiophenein are purchased from 1-material. PBDB-T and F-M are dissolved in *ortho*-xylene (1:1 ratio, 17 mg/mL). The mixture is heated to 100 °C and is stirred overnight in the glovebox. A total of 1.5 h before using the solution, 0.2% of 1,8-diiooctane (DIO) is added. The spin-coating is done dynamically with speeds of 800, 1000, 1400, 2000, and 3000 rpm for 40 s to obtain different active-layer thicknesses. Active-layer thicknesses are estimated with capacitance measurements as described below. The process ends with evaporation of 8 nm of molybdenum oxide (MoO_*x*_) and ∼100 nm silver (Ag) under high vacuum (∼5 × 10^–7^ mbar). A shadow mask defined the device area *A* of 0.06 and 0.16 cm^2^.

### Device Characterization

For the *J*–*V* measurements with irradiation under 1 sun, we use a solar simulator from LOT Quantum Design and a Keithley 2450 as the source meter. The samples are measured in a sealed box. Hence, the incident light is reflected two times at the glass window, so that current densities are adjusted with additional +8%. For estimation of active-layer thicknesses, capacitance measurements are made with a potentiostat Interface 1000 by the company Gamry Instruments. The capacitance at high negative voltages (−3 V) is measured, and a permittivity ε_r_ of 3.9 is used. Intensity-dependent measurements are done with a warm white LED (CXA3050-0000-000N0YU227H) by the company Cree. According to the manufacturer, the LED has a color temperature of 2700 K. The spectral irradiance measurements are done with the array spectrometer CAS 140 with an integrating sphere of the company Instrument Systems. The quantum efficiency is measured with a home-made setup. A BENTHAM 605 provides the halogen and xenon lamp with power. The monochromator TM300 of the company BENTHAM has a symmetric Czerny-Turner geometry and uses diffraction gratings. As for the *J*–*V* measurements, the current densities from *Q*_e_ measurements are also corrected with 8% refraction losses.

## Supporting Information Available

(Figure S1) Performance parameters for different LED spectra; (Figure S2) absolute spectral irradiances; (Figure S3) irradiance *E*_e_ plotted against the illuminance *E*_v_ for all currents and filters; (Note 1) effect of reflective lids on the input-power density; (Table S1) input-power densities and illuminances of all LED currents; (Figure S4) thickness-dependent performance parameters at 1 sun; (Table S2) device performance with a LED-driving current of 45 mA and an OD 2 filter; (Figure S5) intensity-dependent performance; (Note 2) critical resistance; (Figure S6) performance parameters at 1 sun conditions versus *R*_p,dark_; (Table S3) parameters for the simulation in ASA; (Figure S7) simulated *J*–*V* curves and simulated shifted *J*–*V* curves; (Note 3) fitting procedure; (Figure S8) examples fit; (Figure S9) photo-shunt resistance for more samples; (Figure S10 and Note 4) discussion shifted *J*–*V* curves; (Figure S11) performance versus *R*_p,photo_for more samples; (Note 5) calculation of FF_sh_; (Figure S12) *J*_0,*V*oc_ for low intensities; (Figure S13) discussion splitting of *J*_0,*V*oc_(*d*) at different intensities; (Figure S14) simulated *J*_0,*V*oc_, *n*_id,l_ and *V*_oc_(*d*); and (Note 6) derivation of *J*_0,*V*oc_ (PDF)
