# Supplementary material for: Understanding the Thickness and Light-Intensity Dependent Performance of Green-Solvent Processed Organic Solar Cells
Source: ACS Mater Au. 2023 Jan 25;3(3):215–30. doi: 10.1021/acsmaterialsau.2c00070 (PMC10176617; doi:10.1021/acsmaterialsau.2c00070)
Supplement: Supplementary file 1 — mg2c00070_si_001.pdf [file mg2c00070_si_001.pdf]

Supporting Information

Understanding the thickness and light- intensity dependent performance of green-solvent processed organic solar cells

Dana Lübke, Paula Hartnagel, Markus Hülsbeck and Thomas Kirchartz*

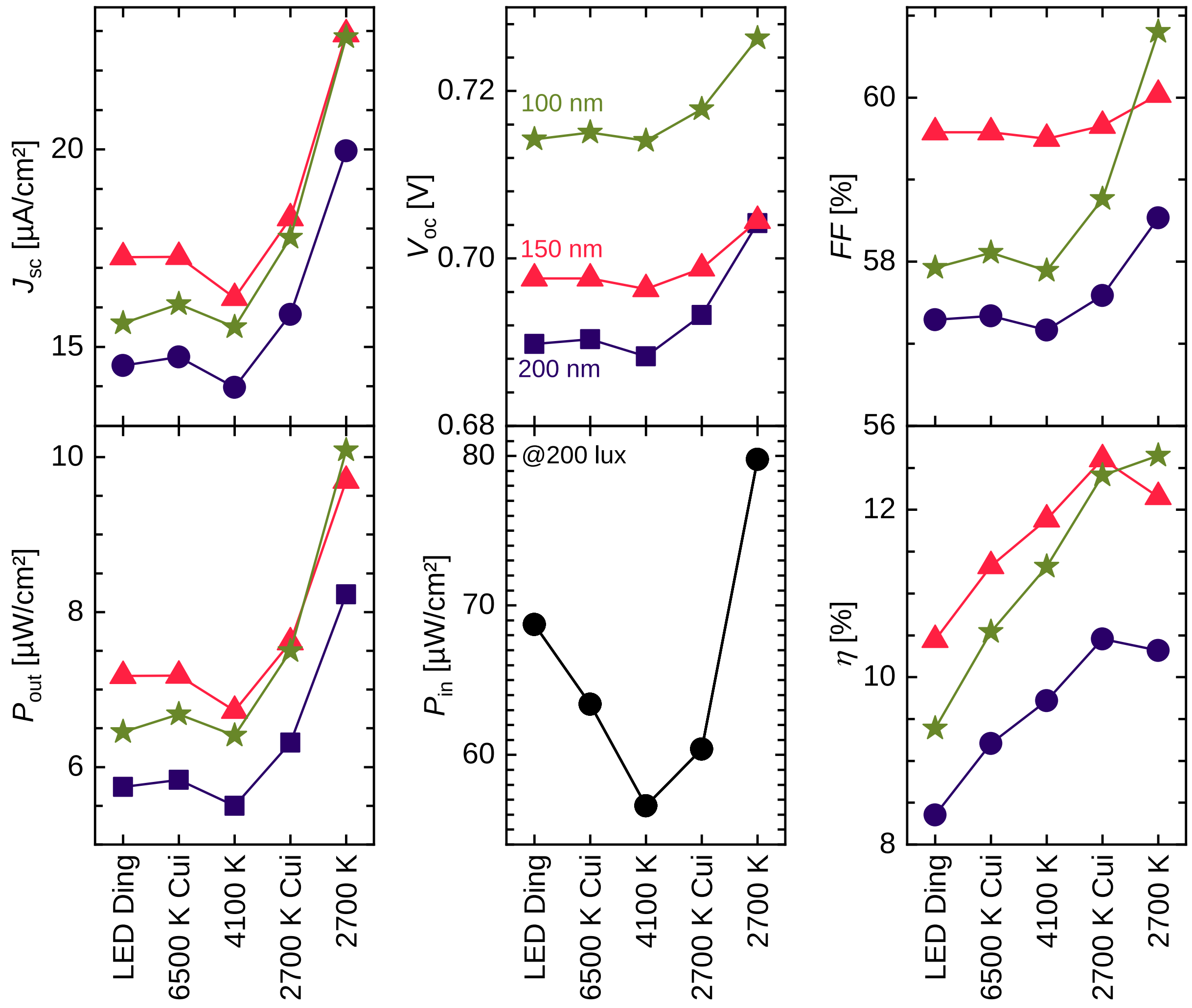

Figure S1. Performance parameters of the devices with different active-layer thicknesses with A = 0.16 cm^2 for different LED spectra and an illumination of 200 lux according to the analysis presented in Ref 1. Best efficiencies are reached for LEDs with a 2700 K spectrum due to the maximized overlap of the device's external quantum efficiency $Q_{e,PV}$ and the spectral irradiance $E_{e,\lambda}$ of the LED. Note, that compared to the measured/interpolated J_{sc}s (Figure 7, ~30 µA/cm^2 at 200 lux), strongly reduced J_{sc}s are found in this calculation. As we measured our data close to 200 lux with an OD 2 Filter, LED spectra are strongly red-shifted and the input-power density is ~140 µW/cm^2. Hence, the measured data shows increased J_{sc}s. This example illustrates again, that device performance can only be evaluated correctly if corresponding LED spectra and accurately measured input-power densities are shown.

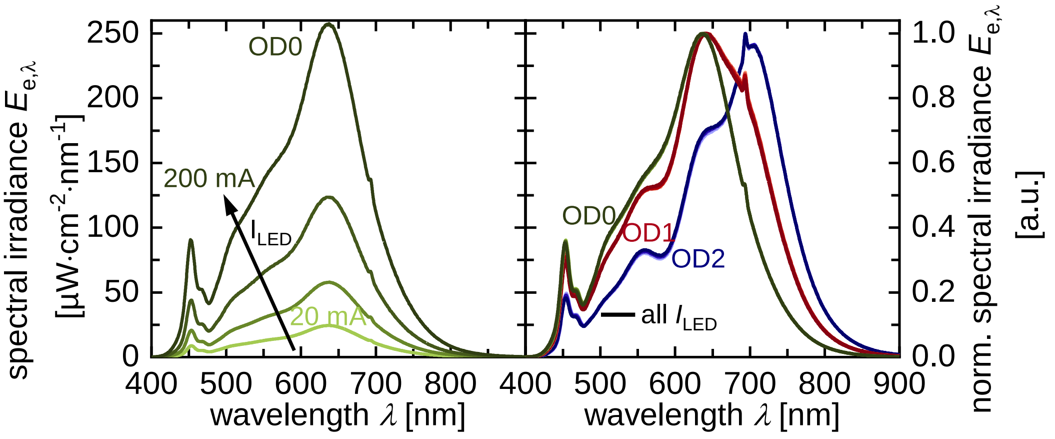

Figure S2. Absolute spectral irradiances of a 2700 K LED for different LED currents I_{LED} and normalized spectral irradiances for the used OD 2 (blue), OD 1 (red) filter and without filter (green, OD 0). Note, that the normalized spectral irradiances are plotted for all I_{LED}, showing that the spectra are constant within the used LED current range.

Note 1: Effect of reflective lids on the input-power density

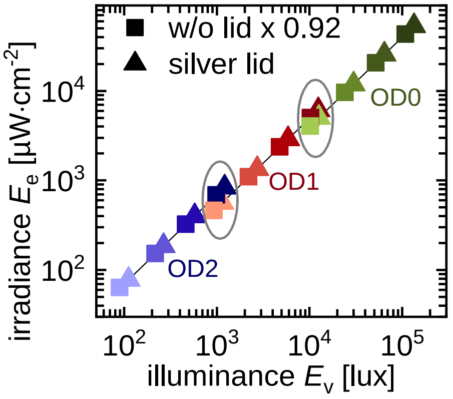

Figure S3: Irradiance E_{e} plotted against the illuminance E_{v} for all currents and filters used. Data measured without a lid (with lid) are depicted with squares (triangles).

Usually, organic solar cells are measured in a sealed sample box to be protected from degradation. Cui et al. pointed out that the use of aperture masks with a reflecting surface like stainless-steel can lead to higher J_{sc} by absorption of scattered light.[2] Here we

demonstrate, the impact of silver lids on the measured irradiances. We performed absolute spectral irradiance measurements of our used LED without a lid and with a windowed stainless-steel lid at the exact position of the solar cell. In order to compare both measurements, the irradiance without a lid is corrected by –8 %, as the light entering the sample box is refracted at the glass window two times. In Figure S3 the irradiances are plotted against the illuminances. The irradiance (and therefore also the illuminance) measured with the silver lid (squares) are $\sim 23 \pm 0.8$ % higher compared to the corrected irradiance without a lid (triangles) for the whole intensity range. We assume that a black lid is not refracting any light. Hence, for samples measured in boxes with black lids we use the irradiances/illuminances without lid corrected by the –8 % refractions losses. Consequently, if comparing samples measured with a silver lid and a black lid, silver lid measurements will be shifted towards higher illuminances/irradiance. The exact values of the whole measuring range are depicted in Table S1.

Table S1. Input-power densities and illuminances of all LED currents and filters measured with an integrating sphere at the exact position of the solar cell with and without a silver lid.

		with lid		w/o lid x 0.92	
Filter	I_{LED} [mA]	P_{in} [W/m^2]	E_{v} [lux]	P_{in} [W/m^2]	E_{v} [lux]
OD 2	20	0.78	112	0.64	90
OD 2	45	1.86	266	1.52	216
OD 2	95	4.01	578	3.24	462
OD 2	200	8.40	1218	6.87	987
OD 1	20	5.68	1148	4.61	928
OD 1	45	13.5	2732	11.0	2212
OD 1	95	29.0	5891	23.7	4789
OD 1	200	61.1	12493	50.3	10267
-	20	50.2	12640	40.5	10185
-	45	119	30004	96.0	24170
-	95	255	64411	206	51886
-	200	532	134761	429	108416

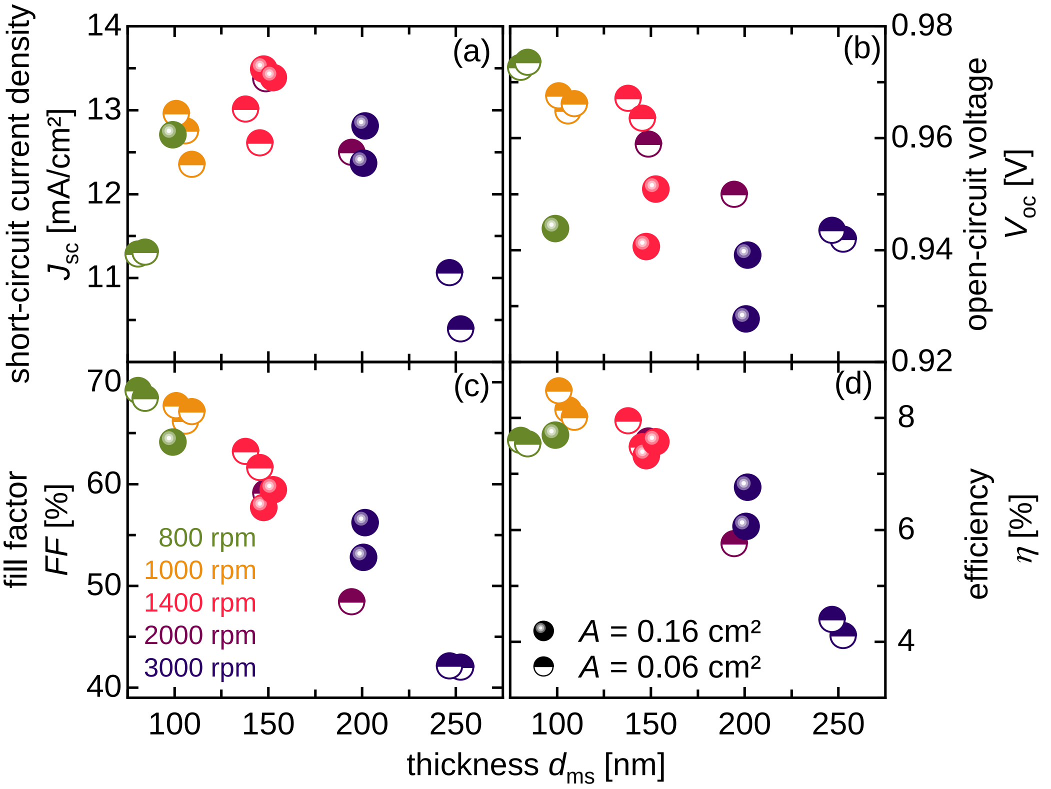

Figure S4. Thickness-dependent performance parameters of PBDB-T:F-M organic solar cells at 1 sun conditions for cell areas of $A = 0.16\ cm^2$ (full circles) and $A = 0.06\ cm^2$ half circles.

Table S2. Device performance of OPV devices (A = 0.16 cm^2 and A = 0.06 cm^2) fabricated for this work under illumination with the 2 OD filter @45 mA (resulting in ~200 lux)

d[a] [nm]	active area A [cm^2]	illuminance [lux]	J_{sc} [μA/cm^2]	$J_{sc,cal}$[b] [μA/cm^2]	V_{oc} [V]	FF [%]	$\boldsymbol{P}_{\mathbf{out}}$ [μW/cm^2]	η [%]
**100**	0.16	216	35.6	31.2	0.74	64.2	17.0	11.2
**150**	0.16	216	35.0	35.3	0.71	60.6	15.2	10.0
**200**	0.16	216	36.0	36.5	0.73	61.3	16.2	10.6
**85**	0.06	265	36.7	-	0.55	32.4	6.6	3.5
**110**	0.06	265	40.4	-	0.77	53.9	16.7	9.0
**140**	0.06	265	38.6	-	0.75	49.1	14.2	7.7
**150**	0.06	265	39.9	-	0.71	40.6	11.6	6.2
**250**	0.06	265	42.6	-	0.74	63.0	20.0	10.7

[a] estimated with capacitance-voltage measurements [b] calculated from $Q_{e,PV}$ measurements

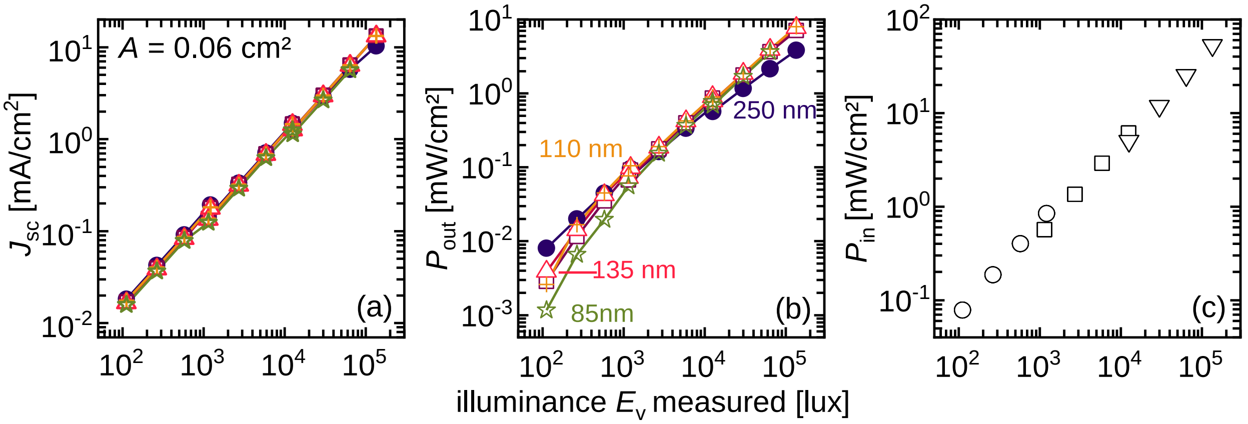

Figure S5. a) Short-circuit current density J_{sc}, b) output-power density P_{out} and c) input-power density P_{in} of the PBDB-T:F-M solar cells with A = 0.06 cm on a double-logarithmic scale.

Note 2: Critical resistance

In the following the critical resistance $R_{\mathrm{p,crit}}$ is derived. The voltage drops over the series resistance and the shunt resistance equal the external voltage $JR_{\mathrm{s}} + J_{\mathrm{p}}R_{\mathrm{p}} - V_{\mathrm{ext}} = 0$, where J_{p} is the current through the shunt resistance, J_{d} is the current flowing through the diode and J is the current flowing through the series resistance with $J = J_{\mathrm{d}} + J_{\mathrm{p}}$. We now consider the worst-case scenario (at the maximum power point), where R_{p} is so small, that all current flows through the shunt and $J = 0$ and $J_{\mathrm{d}} = J_{\mathrm{p}} = J_{\mathrm{mpp}}$. The current is then given by $J_{\mathrm{mpp}} = V_{\mathrm{mpp}}/R_{\mathrm{p}}$ and the critical shunt resistance is $R_{\mathrm{p,crit}} = V_{\mathrm{mpp}}/J_{\mathrm{mpp}}$, which is the minimum shunt resistance at which all current flows through the shunt resistance. Considering $FF = V_{\mathrm{mpp}}J_{\mathrm{mpp}}/V_{\mathrm{oc}}J_{\mathrm{sc}}$ and $J_{\mathrm{mpp}} \approx J_{\mathrm{sc}}$, we arrive at $R_{\mathrm{p,crit}} = FF\ V_{\mathrm{oc}}/J_{\mathrm{sc}}$.

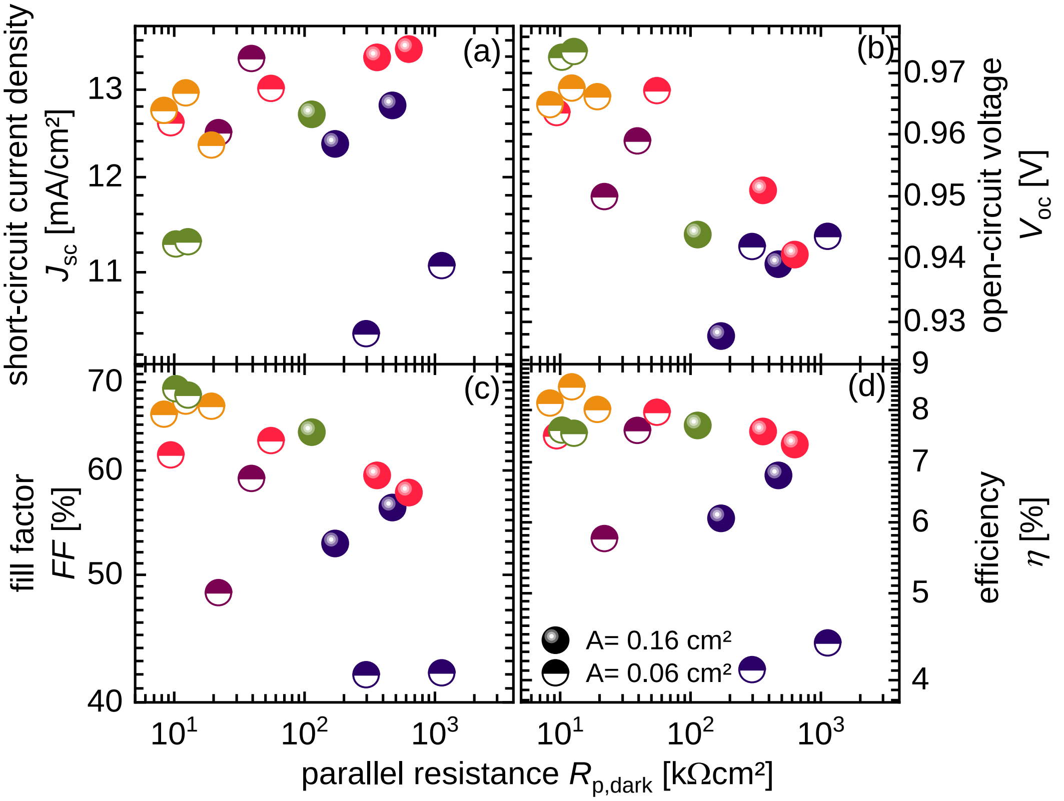

Figure S6. Performance parameters of PBDB-T:F-M organic solar cells at 1 sun conditions for cell areas of A = 0.16 cm^2 (full circles) and A = 0.06 cm^2 (half circles) plotted against the dark parallel resistance $R_{\mathrm{p,dark}}$.

Table S3. Parameters for the simulation of a generic organic solar cell in ASA using the effective-medium model[3,4]. For simplicity, the generation rate G was kept constant over the entire volume. It was defined in such a way that the generation-current density is independent of the active-layer thickness as $G = J_{gen}/(qd)$. In the case of Shockley-Read-Hall recombination, we assumed a distribution of acceptor-like trap states around the center of the bandgap. The recombination mechanisms stated below are the only recombination pathways allowed in the simulations. If not mentioned otherwise, no external resistances are included.

Parameter	Symbol	SRH	direct	Direct + SRH
Effective density of states conduction band/valence band	$N_{CB/VB}$	10^{19} cm^{-3}	10^{19} cm^{-3}	10^{19} cm^{-3}
Effective bandgab energy	$E_{g,eff}$	1.5 eV	1.5 eV	1.5 eV
Injection barrier front/back contact	$\Phi_{bf/bb}$	0.1 eV	0.1 eV	0.1 eV
Electron/hole mobilty	$\mu_{e/h}$	5×10^{-4} cm^2/(Vs)	5×10^{-4} cm^2/(Vs)	5×10^{-4} cm^2/(Vs)
Relative dielectric permittivity	ε_r	4	4	4
Generation-current density	J_{gen}	13.5 mA/cm^2	13.5 mA/cm^2	13.5 mA/cm^2
Direct recombination coefficient	k_{dir}	0	5×10^{-10} cm^3/s	5×10^{-10} cm^3/s
Trap density	N_t	10^{15} cm^{-3}	0	3×10^{14} cm^{-3}
Width of Gaussian trap distribution	ΔE	0.1 eV	-	0.1 eV
Trap energy	E_t	0.75 eV	-	0.75 eV
Capture rate electrons/holes	$b_{e/h}$	5×10^{-9} cm^3/s	-	5×10^{-9} cm^3/s

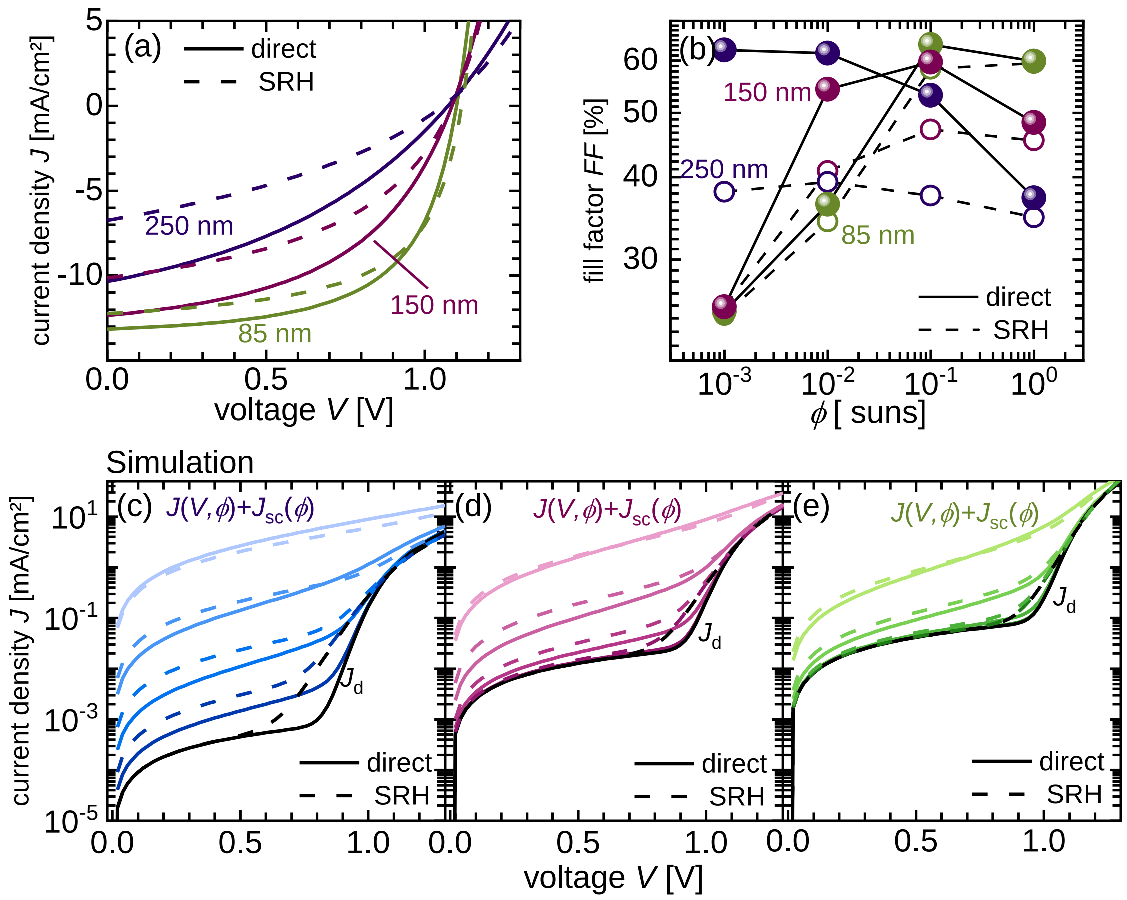

Figure S7. a) Simulated JV curves at 1 sun. b) FFs of the simulated JV curves plotted against the intensity. Simulation shifted JV curves for devices with an active-layer thickness of a) 250 nm b)150 nm and c) 85 nm for direct (solid lines) and SRH (dashed lines) recombination. No mayor differences between direct and SRH recombination can be seen.

Note 3: Procedure for fitting *JV* characteristics

To obtain the parasitic resistances, a simple equivalent-circuit model was fitted to the measured JV characteristics in the dark. The current through the equivalent-circuit with one diode, one shunt resistance $R_{\mathrm{p,dark}}$ and one series resistance R_{s} can be described as

$$J = J_0\left(\exp\left(\frac{q(V_{\mathrm{ext}}-JR_{\mathrm{s}})}{n_{\mathrm{id,d}}kT}\right)-1\right)+\frac{V_{\mathrm{ext}}-JR_{\mathrm{s}}}{R_{\mathrm{p,dark}}}-J_{\mathrm{sc}}, \qquad \text{(S1)}$$

where J_0 is the saturation-current density, $n_{id,d}$ the ideality factor, V_{ext} the external voltage, q the elementary charge, k Boltzmann's constant and T is the temperature of the solar cell. Firstly, the current density J according to Equation S1 is calculated with average starting values for J_0, $n_{id,d}$, R_s and $R_{p,dark}$. The voltage axis of the original measured data is used as the internal voltage V_{int} (instead of $V_{ext} - JR_s$ in Equation S1), and subsequently the voltage drop over R_s is added to the internal voltage to describe the external voltage of the circuit $V_{ext} = V_{int} + JR_s$. Then the sum over all logarithmic differences of the calculated and the measured current values $(\sum_i |\log\left(\frac{J_i}{J_{i,measured}}\right)|)$ is minimized by the *MATLAB* function *fminsearch*. This results in a first approximation of the variables J_0, $n_{id,dark}$, R_s and R_p. The procedure is repeated iteratively until the error is minimized.

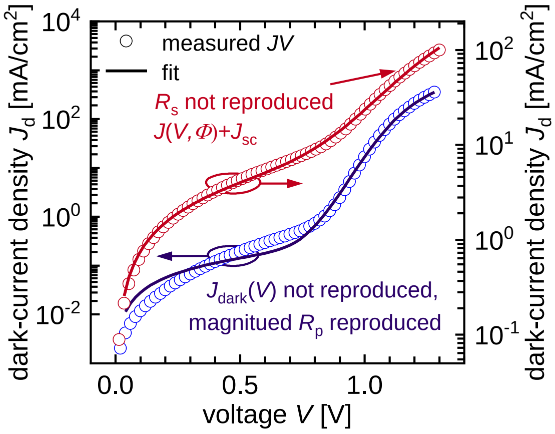

Figure S8. Exemplary JV characteristics (circles) and corresponding fit (solid lines). For devices with non-linear shunts, the JV dependence is not reproduced accurately (blue). In some cases, fitting the $J(V, \Phi) - J_{sc}$ data leads to underestimated R_s as the diode region is less pronounced (red).

In general, with the simple equivalent-circuit a good fit is achieved, but in some cases, where the shunt is non-linear, the small-voltage region is not reproduced accurately. Nevertheless, as the $R_{\mathrm{p,dark}}$ is by the magnitude of data, and not by the exact JV dependence, the fitting procedure is sufficient to determine the $R_{\mathrm{p,dark}}$. For the fitting procedure of the $J(V,\Phi) - J_{\mathrm{sc}}(\Phi)$ data the R_{s} is not reproduced well for high intensities, as the region dominated by the diode is less pronounced.

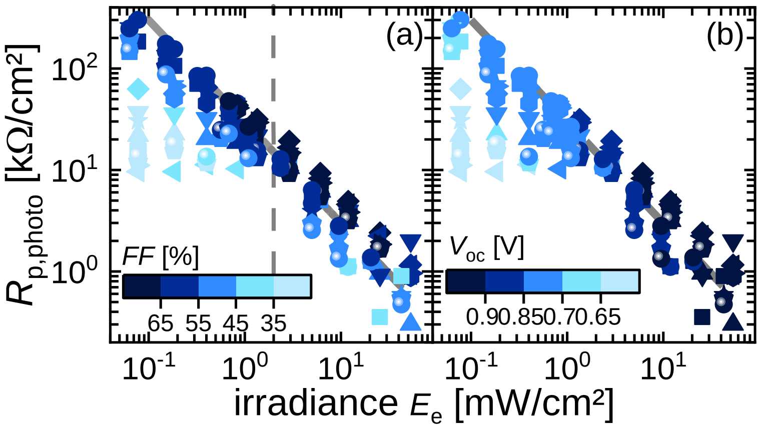

Figure S9. Photo-shunt resistances of the measured shifted JV characteristics versus the irradiance for a wide variety of samples. Dark blue points correspond to high a) FF and b) V_{oc} and light blue points correspond to low a) FF and b) V_{oc}.

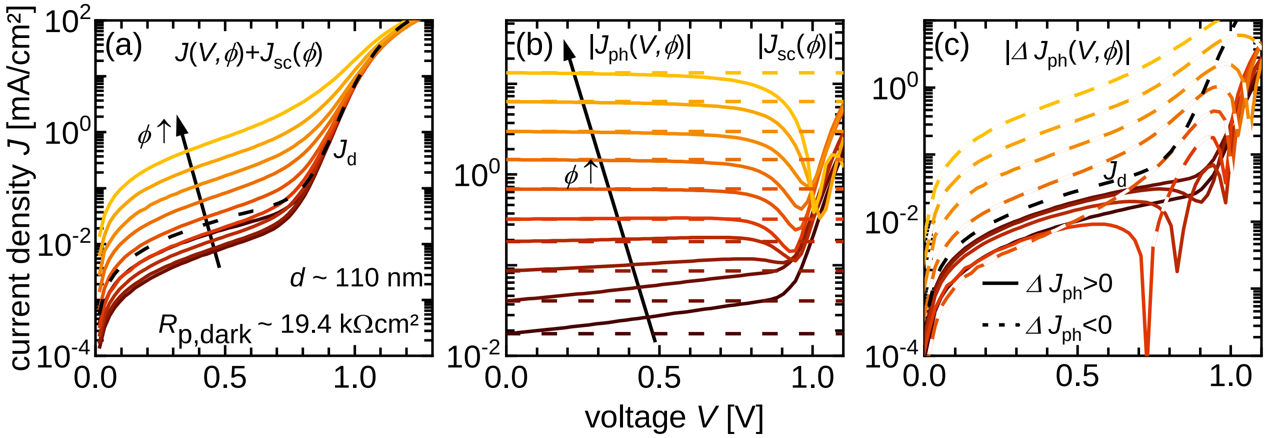

Figure S10. a) Shifted JV curves (red to yellow lines) for a device with 110 nm active-layer thickness and dark JV curve (dashed black line). b) Absolute photocurrent density $|J_{ph}(V,\Phi)| = J(V,\Phi) - J_{dark}(V)$ (red to yellow solid lines) and absolute short-circuit current density $|J_{sc}(\Phi)|$ (red to yellow dashed lines). c) Absolute values of the difference $|\Delta J_{ph}(V,\Phi)| = |J_{ph}(V,\Phi)| - |J_{sc}(\Phi)|$ (red to yellow lines). Solid lines indicate $\Delta J_{ph}(V,\Phi) > 0$ (low intensities) and dashed lines indicate $\Delta J_{ph}(V,\Phi) < 0$ (high intensities). The black dashed line is the dark JV curve.

Note 4: Photocurrent

For the region of high intensities $|J_{ph}(V,\Phi)| < |J_{sc}(\Phi)|$ holds true and performance is maintained. The extraction of charge carriers is lower under a certain voltage than in the short-circuit condition. The term $\Delta J_{ph}(V,\Phi) < 0$ (dashed lines in Figure S10c)) and thus, the shifted JV curves $J(V,\Phi) + J_{sc}(\Phi) = J_{dark}(V) - \Delta J_{ph}(V,\Phi)$ increase with increasing intensities. For lower intensities in some cases, we see $|J_{ph}(V,\Phi)| > |J_{sc}(\Phi)|$ and the term $\Delta J_{ph}(V,\Phi) > 0$ (solid lines in Figure S10c)). Here, the shifted JV curves fall below the dark JV curve. Usually, one would expect the photocurrent under a certain voltage to be lower than the current in the short-circuit condition, so this behavior is still under debate, but can be discussed by considering the spatial generation and recombination rates. The photocurrent and short-circuit current density can be expressed as the difference between charge

generation and charge recombination $J_{\mathrm{ph}}(\Phi, V) = q\int (G(x, \mathit{\Phi}, V) - R(x, \mathit{\Phi}, V))\, dx$ and $J_{\mathrm{sc}}(\Phi, 0) = q\int (G(x, \mathit{\Phi}, 0) - R(x, \mathit{\Phi}, 0))\, dx$. With the observation of the photocurrent being higher than the short-circuit current density we can argue $|G(V, \mathit{\Phi}) - \mathrm{R}(V, \mathit{\Phi})| > |G(0, \mathit{\Phi}) - \mathrm{R}(0, \mathit{\Phi})|$. If we assume that the recombination under a certain voltage is higher than a zero volt $\mathrm{R}(V, \mathit{\Phi}) > \mathrm{R}(0, \mathit{\Phi})$, the expression above leads us to $G(V, \mathit{\Phi}) > G(0, \mathit{\Phi})$. We cannot think of any case where the generation rates under a certain voltage should be higher compared to short-circuit. On the other hand, if we assume that the generation rate is independent of the applied voltage, the expression above will result in $\mathrm{R}(V, \mathit{\Phi}) < \mathrm{R}(0, \mathit{\Phi})$, which also seems to be an unrealistic relation. Up till now we cannot explain our data showing $\left|J_{\mathrm{ph}}(V, \mathit{\Phi})\right| > |J_{\mathrm{sc}}(\mathit{\Phi})|$.

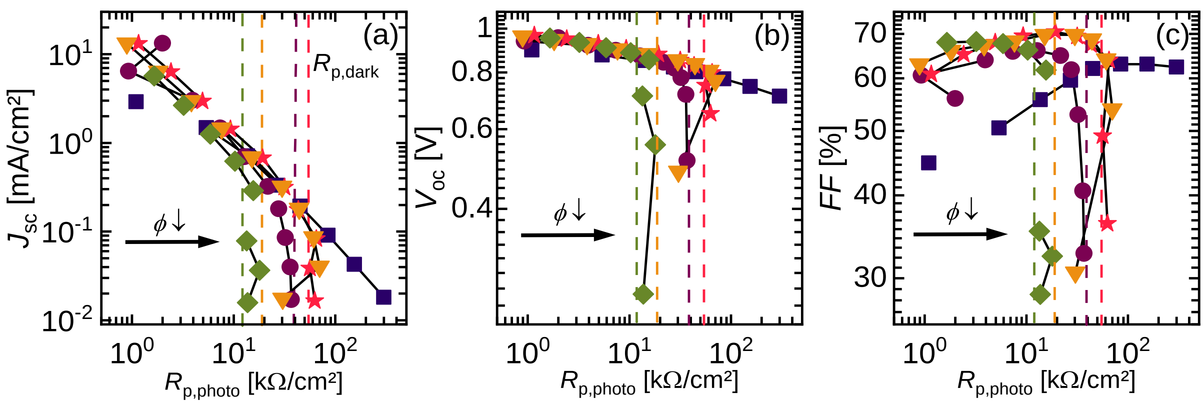

Figure S11. a) Short-circuit current density J_{sc}, b) the open-circuit voltage V_{oc} and c) fill factor FF plotted against the photo-shunt resistance $R_{p,photo}$ on a double-logarithmic scale for the devices with different thicknesses and A = 0.06 cm^2. The dashed lines indicate the dark-shunt resistance $R_{p,dark}$.

Note 5 Empirical equation for fill factors by Green et al. according to Ref. 5 (for devices suffering from shunt and series resistances losses)

$$FF_{sh} = FF_s\left(1 - \frac{(v_{oc}+0.7)FF_s}{v_{oc}\, r_{sh}}\right)$$

with

$$FF_s = FF_0(1 - r_s)$$

and

$$FF_0 = \frac{v_{oc} - \ln(v_{oc}+0.72)}{v_{oc}+1} \tag{S2}$$

Here v_{oc} is the normalized open-circuit voltage $v_{oc} = qV_{oc}/n_{id,photo}kT$, r_s is the normalized series resistance and r_{sh} is the normalized shunt resistance $r_{sh} = R_{p,photo}\, J_{sc}/V_{oc}$. The ideality factor was determined by fitting of the shifted JV curves.

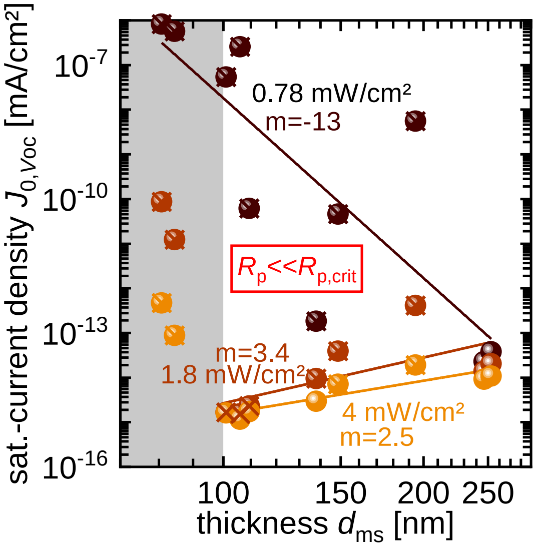

Figure S12. Saturation-current density $J_{0,V\mathrm{oc}}$ at V_{oc} plotted against the active-layer thickness on a double-logarithmic scale for small intensities and for devices with an active area of A = 0.06 cm². For intensities < 8 mW/cm², the V_{oc} is decreased by leakage currents through the shunt resistance (crossed spheres). Therefore, the $J_{0,V_{\mathrm{oc}}}$ is increased and the values should not be interpreted in terms of the method by Zonno et al..

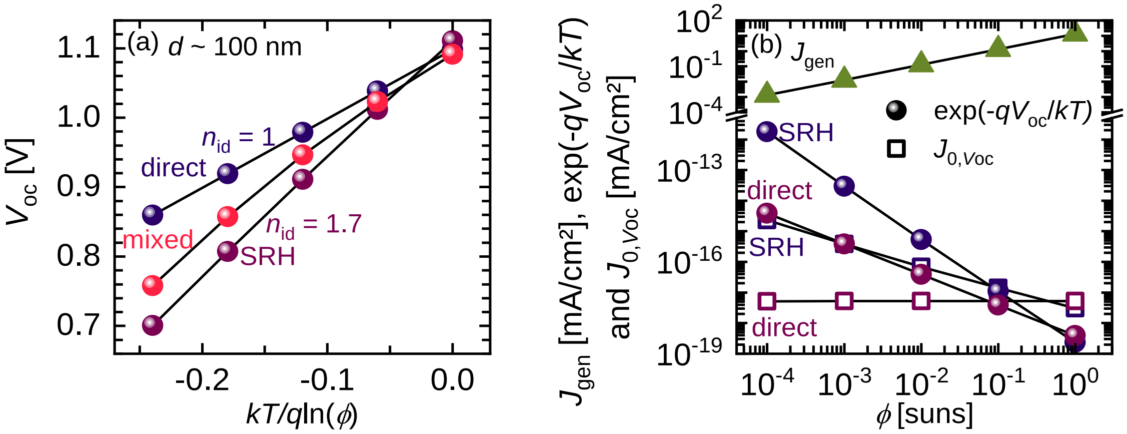

Figure S13. a) V_{oc} plotted against $kT/q\,\ln(\Phi)$ for the simulated cases of direct, mixed and SRH recombination at an active-layer thickness of ~100 nm. The slope indicates the ideality factor $n_{\text{id,l}}$. For SRH recombination the V_{oc} is increasing faster with increasing light intensity compared to the case of direct recombination as the ideality factor is higher. b) Different terms of Equation 8 are compared for direct and SRH recombination and for different intensities at a constant thickness of ~100 nm on a double-logarithmic axis. The generated current J_{gen} (green triangles) is increasing linearly with the intensity (slope of 1 on double-logarithmic axis). The exponential term in Equation 8 $\exp(-qV_{\text{oc}}/kT)$ (spheres) is displayed for direct (purple) and SRH (blue) recombination. If we plug in $V_{\text{oc}} = n_{\text{id}}kT/q\,\ln(\Phi)$ into the exponential term of Equation 8, we get to $\exp(-qV_{\text{oc}}/kT) = \Phi^{-n_{\text{id}}}$. Consequently, the slope on the double-logarithmic axis $d\ln(\exp(-qV_{\text{oc}}/kT))/d\ln(\Phi) = -n_{\text{id}}$. This means that for direct recombination where $n_{\text{id}} = 1$, the exponential term is decreasing as fast as the J_{gen} is increasing, so that the $J_{0,Voc}$ is constant with the intensity. On the other hand, for SRH recombination, the V_{oc} is increasing faster with increasing intensity (with a slope of ~1.7). Hence, the exponential term is decreasing faster compared to J_{gen} resulting in a decreasing $J_{0,Voc}$. This discussion shows that for increasing n_{id} the $J_{0,Voc}$ is decreasing.

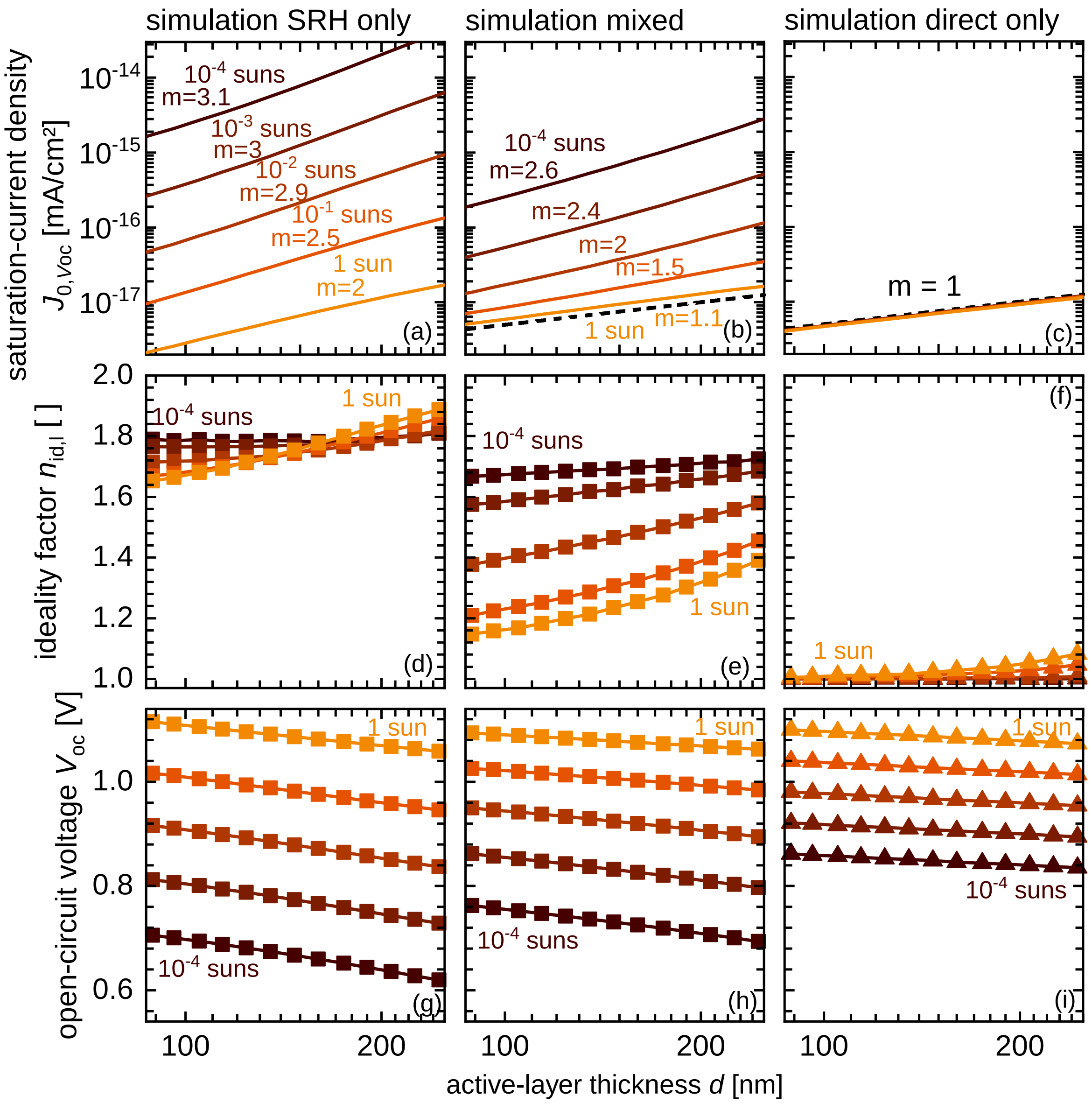

Figure S14. Simulated saturation-current density $J_{0,Voc}$ at V_{oc} plotted against the active-layer thickness on a double-logarithmic scale for intensities between 10^{-4} and 1 sun for a) SRH recombination, b) for mixed recombination and c) for direct recombination. Ideality factor $n_{id,l}$ (determined from $V_{oc}(J_{sc})$) for d) SRH recombination, e) for mixed recombination and e) for direct recombination. Open-circuit voltage V_{oc} plotted against the thickness for g) SRH recombination, h) for mixed recombination and i) for direct recombination.

Note 6: Derivation of $J_{0,V\mathrm{oc}}(d)$

For the open-circuit condition and for sufficient negative biases, where $J_{\mathrm{gen}} \approx J_{\mathrm{sc}}$, the measured $J_{0,V\mathrm{oc}}$ is described by

$$J_{0,V\mathrm{oc}} = J_{\mathrm{sc}} \exp\left(-\frac{qV_{\mathrm{oc}}}{k_{\mathrm{b}}T}\right). \tag{S3}$$

On the other hand, the recombination-current density can be described as the integral over the spatially recombination rates $J_{\mathrm{rec}} = q\int R(x)\,dx$ with $R_{\mathrm{SRH}} = n/\tau_{\mathrm{eff}}(n)$ for traps in the middle of the band gap. Here, n is the concentration of free electrons and τ_{eff} is the effective lifetime, assuming $n = p$ and equal lifetimes for electrons and holes. Hence, for SRH recombination the recombination-current density is $J_{\mathrm{rec}} = qn/\tau_{\mathrm{eff}}(n)\ d$. Alternatively, we can describe the dependence of τ_{eff} on n by defining a constant lifetime and express the dependency on n with a voltage-dependent term $R_{\mathrm{SRH}} = \frac{n}{\tau_{\mathrm{eff}}(n)} = \frac{n_{\mathrm{i}}}{\tau_0}\exp\left(\frac{qV}{n_{\mathrm{id}}kT}\right)$, where n_{i} is the intrinsic charge-carrier density and n_{id} is the ideality factor. Then, the recombination-current density is

$$J_{\mathrm{rec}}(V_{\mathrm{oc}}) = J_{\mathrm{sc}} = \frac{qn_{\mathrm{i}}d}{\tau_0}\exp\left(\frac{qV_{oc}}{n_{\mathrm{id}}kT}\right) = J_0\ \exp\left(\frac{qV_{oc}}{n_{\mathrm{id}}kT}\right). \tag{S4}$$

Plugging in Equation S4 into Equation S3 leads us to

$$J_{0,V\mathrm{oc}}(V_{\mathrm{oc}}) = qn_{\mathrm{i}}d/\tau_0\ \exp\left(\frac{qV_{\mathrm{oc}}}{kT}\left(\frac{1}{n_{\mathrm{id}}} - 1\right)\right) \tag{S5}$$

Note, that in the case of direct recombination of free charges, the recombination rate is given by $R_{\mathrm{dir}} = k(np - n_i^2)$. Additionally, we assume $np \gg n_{\mathrm{i}}^2$ and $np = n_{\mathrm{i}}^2\exp(qV/kT)$. As the voltage dependence cancels out, $J_{0,V\mathrm{oc}}$ simplifies to $J_{0,V\mathrm{oc}} = qkn_{\mathrm{i}}d$, which is not a function of the light intensity and constantly increases with thickness with a slope $m = 1$.

Now, we consider the thickness-dependent quantities in Equation S5. The V_{oc} changes with the thickness basically influenced by the thickness dependence of J_{sc} and J_0 as $V_{\mathrm{oc}} \propto \ln(J_{\mathrm{sc}}/J_0)$. The J_0 is linearly increasing with the thickness (see Equation S4). According to Lamber-Beer, the absorptance is increasing exponentially with increasing thickness until all light is absorbed ($a = 1 - \exp(-2\alpha d)$), with a being the absorptance and α being the effective absorption coefficient). Hence, for small thicknesses where $J_{\mathrm{sc}} \propto d$, V_{oc} is constant with thickness. Once the solar cell is thick enough to absorb all incoming light, J_{sc} saturates and is not a function of the thickness (neglecting interference effects). Then, the J_0 determines the thickness dependence of V_{oc}, scaling with $-\ln(d)$. Our simulations also show, that V_{oc} is proportional to $-\ln(d)$ (see Figure S14g,h,i). Hence, we can parametrize the open-circuit voltage as

$$V_{\mathrm{oc}} = V_{\mathrm{oc}}(d_{\mathrm{min}}) - \beta kT/q \ln(d/d_{\mathrm{min}}), \tag{S6}$$

with β being the positive linear slope $\beta = q\, \mathrm{d}V_{\mathrm{oc}}/(kT \mathrm{d}\ln(d))$ if V_{oc} is plotted against $\ln(d)$ and with d_{min} being the minimum thickness. Following Equation S5 and Equation S6 and regarding only the thickness-dependent quantities leads us to

$$J_{0,V\mathrm{oc}}(d) = C\, d^{1+\beta\left(1-\frac{1}{n_{\mathrm{id}}}\right)}$$

$$\text{with } C = \frac{q n_{\mathrm{i}}}{\tau} \exp\left(\frac{q}{kT} V_{\mathrm{oc}}(d_{\mathrm{min}}) \left(\frac{1}{n_{\mathrm{id}}} - 1\right)\right) \frac{1}{d_{\mathrm{min}}}^{\beta\left(1-\frac{1}{n_{\mathrm{id}}}\right)}. \tag{S7}$$

Concluding, if the $V_{\mathrm{oc}} \propto \mathrm{const} - \ln(d)$ and the ideality factor n_{id} is only slightly dependent on the thickness the slope m on the double-logarithmic plot of $J_{0,V\mathrm{oc}}$ versus d is $m = 1 + \beta\left(1 - \frac{1}{n_{\mathrm{id}}}\right)$. Hence, if the V_{oc} decreases faster with the active-layer thickness, β and the slope m increase. For the case of dominating direct recombination, no correction factor is needed

and the slope m as well as β is 1 (see Figure S14c). If the amount of SRH recombination is increasing (n_{id} increases), the slope m is increasing (compare the different intensities for the case of mixed recombination mechanisms in Figure S14b).

Note 7: Thickness and intensity dependence of the ideality factor

The ideality factors as shown in Figure S14 (simulation) and Figure 10c (experiment) indicate that ideality factors tend to increase towards lower light intensities or voltages and towards higher thicknesses. Both effects are slightly superimposed as higher thicknesses also lead to reduced open-circuit voltages. We note that this effect can only be present if at least one recombination mechanism is not proportional to np. Otherwise, the ideality factor will be constant at $n_{id} = 1$ for any light intensity and thickness.

To understand the trend in n_{id}, Figures S15 to S17 show the carrier densities, recombination rates and n/p ratios for thin (100nm) and thick (300nm) active layers at open circuit assuming direct recombination (Figure S15), mixed (direct & SRH) recombination (Figure S16) and only SRH recombination (Figure S17). We observe several trends. For direct recombination, the rate is not position dependent. For all other types of recombination, it is position dependent and it often has a peak in the middle of the device. This peak is generally more strongly visible for lower light intensities. As SRH recombination shows higher recombination rates in the case, where $n = p$, we also compare the rates with the n/p ratio. If the n/p ratio changes quite rapidly with position, the peak of the recombination rate in the middle of the device (see Figs. S16 and S17) will be more pronounced. This is the case for lower light intensities, while the peak smears out for higher light intensities and becomes a

nearly flat plateau. This trend can explain why the ideality factor generally gets higher for lower light intensities as long as SRH recombination is involved. The trend with thickness cannot easily be explained in the same manner. We assume that the dominant effect with thickness is that the region with n and p being approximately equal widens due to space-charge effects (band bending, reduced electric field in the middle of the absorber). This widening is more pronounced at higher light intensities as there are more charge carriers and hence more space charge. This trend is consistent with the stronger increase of n_{id} with thickness for higher light intensities seen in Figure S14.

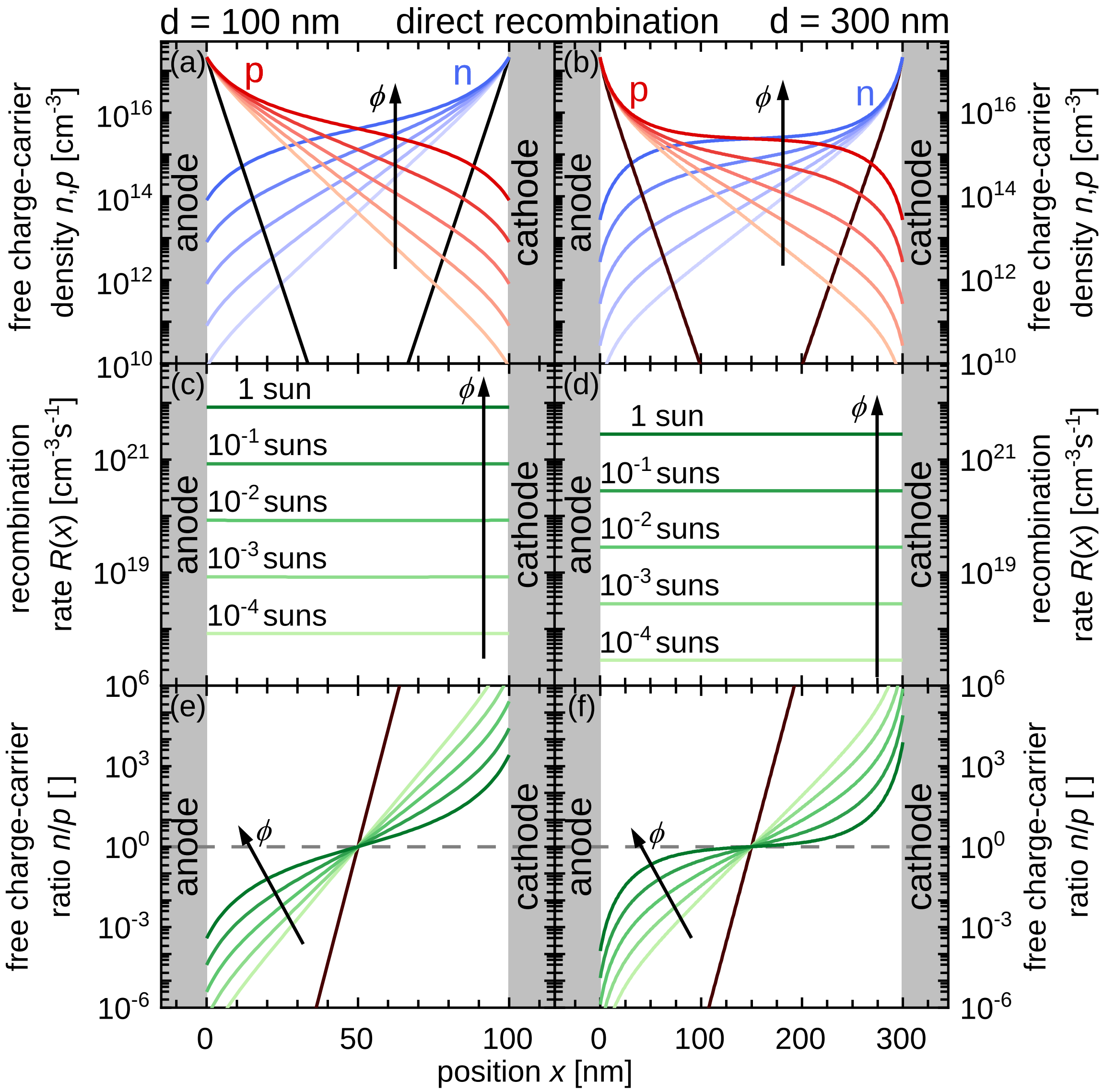

Figure S15. Simulated free charge-carrier densities $n(x)$ and $p(x)$ (a,b), recombination rates $R(x)$ (c,d) and free charge-carrier ratios n/p (e,f) for the case of direct recombination for devices with an active-layer thickness of 100 nm (a,c,d) and 300 nm (b,d,f). The parameters used for the simulation can be found in Table S3.

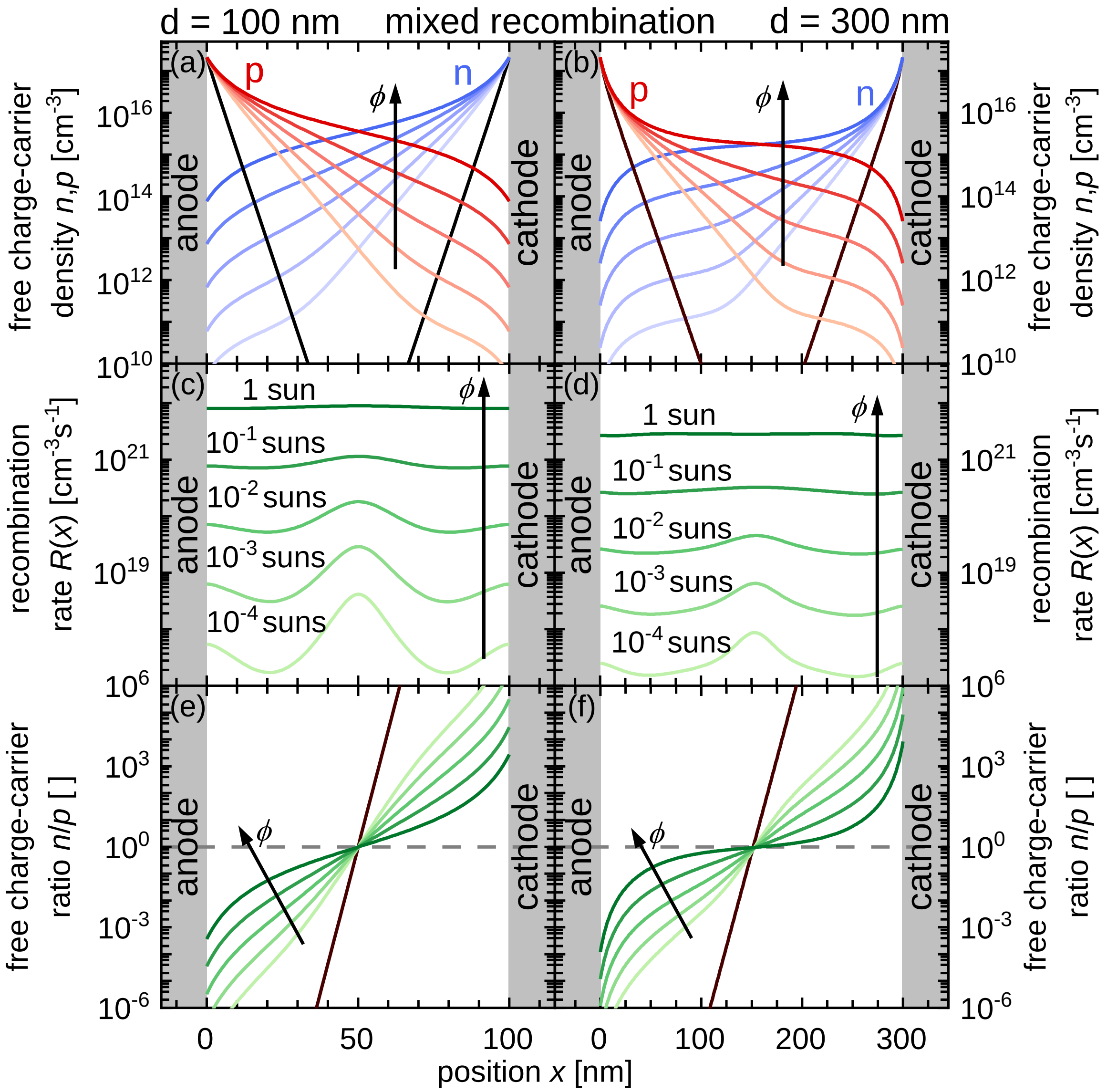

Figure S15. Simulated free charge-carrier densities $n(x)$ and $p(x)$ (a,b), recombination rates $R(x)$ (c,d) and free charge-carrier ratios n/p (e,f) for the case of direct and SRH recombination for devices with an active-layer thickness of 100 nm (a,c,d) and 300 nm (b,d,f). The parameters used for the simulation can be found in Table S3.

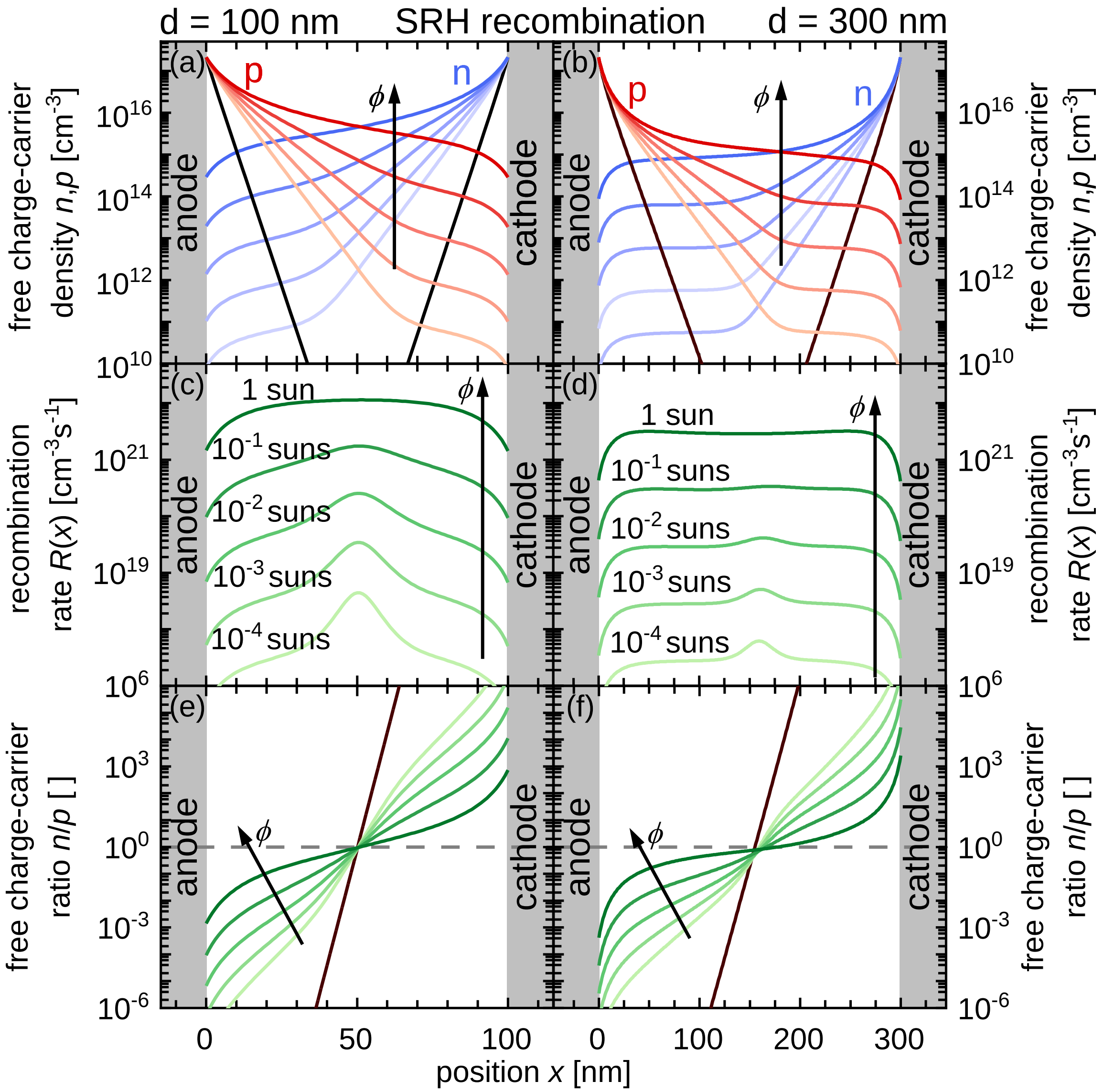

Figure S16. Simulated free charge-carrier densities $n(x)$ and $p(x)$ (a,b), recombination rates $R(x)$ (c,d) and free charge-carrier ratios n/p (e,f) for the case of SRH recombination for devices with an active-layer thickness of 100 nm (a,c,d) and 300 nm (b,d,f). The parameters used for the simulation can be found in Table S3.

REFERENCES

(1) Lübke, D.; Hartnagel, P.; Angona, J.; Kirchartz, T. Comparing and Quantifying Indoor Performance of Organic Solar Cells. *Adv Energy Mater* **2021**, *11* (34), 2101474.

(2) Cui, Y.; Hong, L.; Zhang, T.; Meng, H.; Yan, H.; Gao, F.; Hou, J. Accurate Photovoltaic Measurement of Organic Cells for Indoor Applications. *Joule* **2021**, *5* (5), 1016–1023.

(3) Koster, L. J. A.; Smits, E. C. P.; Mihailetchi, V. D.; Blom, P. W. M. Device Model for the Operation of Polymer/Fullerene Bulk Heterojunction Solar Cells. *Phys Rev B* **2005**, *72* (8), 085205.

(4) Kirchartz, T.; Nelson, J. Device Modeling of Organic Heterojunction Solar Cells. In: Multiscale Modelling of Organic and Hybrid Photovoltaics; Beljonne, D., Cornil, J., Eds.; Topics in Current Chemistry; Springer Berlin Heidelberg: Berlin, Heidelberg, 2014; Vol. 352, pp 279–324.

(5) Green, M. A. Solar Cell Fill Factors: General Graph and Empirical Expressions. *Solid State Electron* **1981**, *24* (8), 788–789.
